# Rescue of Rare CFTR Trafficking Mutants Highlights a Structural Location-Dependent Pattern for Correction

**DOI:** 10.3390/ijms24043211

**Published:** 2023-02-06

**Authors:** Sónia Zacarias, Marta S. P. Batista, Sofia S. Ramalho, Bruno L. Victor, Carlos M. Farinha

**Affiliations:** Biosystems and Integrative Sciences Institute, Faculty of Sciences, University of Lisboa, Campo Grande, 1749-016 Lisboa, Portugal

**Keywords:** Cystic Fibrosis, CFTR, correctors, protein stability, protein trafficking

## Abstract

Cystic Fibrosis (CF) is a genetic disease caused by mutations in the gene encoding the Cystic Fibrosis Transmembrane Conductance Regulator (CFTR) channel. Currently, more than 2100 variants have been identified in the gene, with a large number being very rare. The approval of modulators that act on mutant CFTR protein, correcting its molecular defect and thus alleviating the burden of the disease, revolutionized the field of CF. However, these drugs do not apply to all patients with CF, especially those with rare mutations—for which there is a lack of knowledge on the molecular mechanisms of the disease and the response to modulators. In this work, we evaluated the impact of several rare putative class II mutations on the expression, processing, and response of CFTR to modulators. Novel cell models consisting of bronchial epithelial cell lines expressing CFTR with 14 rare variants were created. The variants studied are localized at Transmembrane Domain 1 (TMD1) or very close to the signature motif of Nucleotide Binding Domain 1 (NBD1). Our data show that all mutations analyzed significantly decrease CFTR processing and while TMD1 mutations respond to modulators, those localized in NBD1 do not. Molecular modeling calculations confirm that the mutations in NBD1 induce greater destabilization of CFTR structure than those in TMD1. Furthermore, the structural proximity of TMD1 mutants to the reported binding site of CFTR modulators such as VX-809 and VX-661, make them more efficient in stabilizing the CFTR mutants analyzed. Overall, our data suggest a pattern for mutation location and impact in response to modulators that correlates with the global effect of the mutations on CFTR structure.

## 1. Introduction

Cystic Fibrosis (CF) is a monogenic disease that affects 100,000 people worldwide, being more prevalent among those of Caucasian descent [1]. CF is caused by mutations in the Cystic Fibrosis Transmembrane Conductance Regulator (CFTR) gene, which encodes a 1480 amino acid-long membrane protein expressed in the apical membrane of epithelial cells [2,3]. CFTR (also called ABCC7) belongs to the ABC transporter superfamily, and evolved towards a specific function as a phosphorylation-activated and ATP-gated ion channel [4], rather than as a transporter. This protein is composed of five domains: two hydrophobic membrane-spanning domains (TMD1 and TMD2) that form the anion-selective pore, two nucleotide-binding domains (NBD1 and NBD2) located in the cytosol, and a unique and largely disordered regulatory domain (R-domain) that connects NBD1 to TMD2 and participates in the control of channel gating via phosphorylation/dephosphorylation of multiple consensus sites [5,6,7,8]. CFTR dysfunction affects the osmotic equilibrium, affecting the epithelial cells lining the airways and leading to the accumulation of a viscous mucus layer that is easily colonized by bacteria, resulting in chronic infection and, eventually, lung failure and death [9]. Parallel to pulmonary hypertension, bronchiolitis obliterans, and interstitial lung, CF is one of the most common diseases that still lead to lung transplantation worldwide [10] with a median survival age around the mid-40s.

More than 2100 gene variants are reported in the Cystic Fibrosis Mutation Database (CFMDB) at http://www.genet.sickkids.on.ca (accessed on 15 November 2022), with a wide range of phenotypes, from classical CF to almost no disease symptoms [11], meaning that the mechanism through which they cause disease is diverse. CFTR mutations have been grouped into functional classes according to the molecular defects that cause disease, and these classes have more recently evolved into theratypes [12]. Class I mutations impair protein production; class II impair protein trafficking; class III impair protein gating; class IV decrease channel conductance; class V decrease protein production; class VI decrease membrane stability, and class VII lead to the absence of full-length mature mRNA. Mutations in classes I, II, III, and VII are usually associated with more severe disease, while those in classes IV, V, and VI are related to milder phenotypes. As individuals with CF may carry different CFTR mutations on the two alleles, this leads to thousands of possible combinations of CF genotypes. Interpretation of the effect of each mutation gets even more complex considering that several mutations exhibit molecular defects characteristic of more than one class.

The clinical features and liability of CFTR mutations are registered in the CFTR2 database (http://www.cftr2.org). With data from more than 89,000 people with CF, this database provides information on the different variants of CFTR, including whether the variant or variant combination causes CF, its variable clinical effects, and whether they have unknown significance or are not CF-causing (current numbers report close to 500 variants classifying more than 400 as CF-causing).

The most common CF-causing mutation is F508del, a deletion of three nucleotides that leads to the deletion of the phenylalanine residue at position 508, which accounts for approximately two-thirds of all alleles in individuals with CF. The remaining third of alleles are heterogeneous and encompass many extremely rare mutations, that occur in less than 0.5% of all individuals with CF [13]. Following F508del, missense mutations represent the largest category of variants associated with CF [14].

In the past 10 years, small molecules that directly target the CFTR defect, called CFTR modulators, have been developed, and they act by enhancing channel activity, protein stability, or folding. The first approved modulator was VX-770 (ivacaftor), a potentiator that corrects CFTR gating/conductance defects and is currently approved for several class III/IV mutations. The corrector lumacaftor (VX-809) was the first compound partially restoring the trafficking of F508del-CFTR [15] that made its way to the clinic as part of Orkambi, a double therapeutic combination with ivacaftor. Subsequently, another double combination of CF modulators (including the corrector tezacaftor and the potentiator ivacaftor) was approved for the treatment of patients with CF. A breakthrough occurred more recently with the approval in the US (and later in Europe) of the triple combination Trikafta /Kaftrio (corrector VX-661 + potentiator VX-770 + next-generation corrector VX-445), focused on people with CF that carry one of almost 200 mutations—which covers 85–90% of people with CF. However, although covering many individuals, modulators are not available to all CF patients due to lack of response, which, for those with rare mutations, may be related to the lack of knowledge on whether they respond or not to modulators.

In this work, we transduced the parental CFBE41o- cell line [16] to generate novel cell lines with heterologous expression of CFTR containing 15 mutations [17] located at either TMD1 or in NBD1 between the signature and the Walker B motifs. Expression and processing were evaluated by Western Blot (WB), with or without treatment with VX-809, VX-661, VX-445, and the double corrector combination VX-661/VX-445. Mutations were also mapped to the human CFTR structure determined by cryoEM [7], and grouped as responders or non-responders to the modulators. Our results show that the mutations analyzed affect CFTR processing (lack or severe reduction in band C—mature form) confirming them as class II. Furthermore, considering their location in CFTR structure, mutations in TMD1 respond to modulators, particularly to the combination VX-661/VX-445, while those located in NBD1 do not. Although focused on CFTR expression and processing (and not channel function), our study confirms the relevance of assessing the response of rare mutations to modulators (in a personalized medicine approach) and suggests the existence of a location-dependent pattern of response to modulators that may contribute to further clarifying the mechanism of action of the correctors. Understanding the molecular and functional consequences of rare CF mutations is fundamental for the adoption of precision therapeutic approaches for such CF patients.

## 2. Results

### 2.1. Rationale and Context for the Mutations Analyzed

In this study, we aim to characterize rare variants, which due to their localization or clinical phenotype are likely to affect CFTR processing and therefore be classified as class II mutations. All variants analyzed are missense—the largest category of variants associated with CF [14].

**Figure 1:**
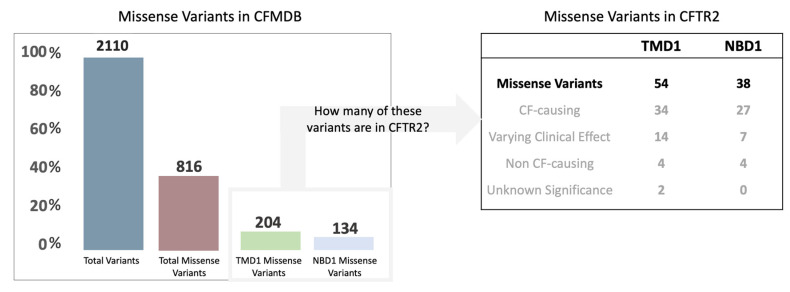
(**left**) Graphical representation of the total number of variants in CFMDB and the number of missense variants localized in TMD1 and NBD1. (**right**) Table indicating the number and classification of missense variants in CFTR2 (for a more detailed table, see Supplementary Table S1).

Among the 2110 variants listed in the CFTR Mutation Database (CFMDB), 816 are missense variants (about 40% of the total registered variants). Focusing on those located in TMD1 and NBD1, there are 204 (about 25% of the total) and 134 (about 15% of the total) missense variants registered, respectively (Figure 1). From these numbers and considering those in CFTR2, 54 are localized in TMD1 (34 reported as CF-causing) and 38 in NBD1 (27 reported as CF-causing).

**Figure 2:**
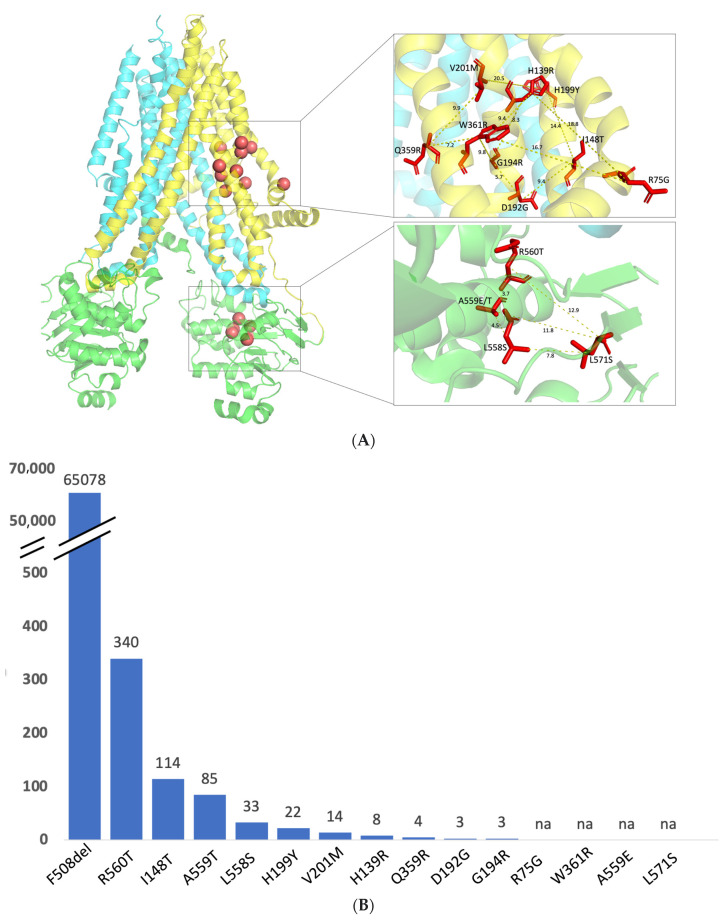
Location and incidence of the variants under study. (**A**) CFTR structure (PDB: 5UAK) with TMD1 in yellow, TMD2 in blue, and NBD1 and NBD2 in green. The red spheres indicate the position of the variants analyzed—R75G, H139R, I148T, D192G, G194R, H199Y, V201M, Q359R and W361R are localized in TMD1, while A559T, A559E, L558S, and L571S are found in NBD1. Insets identify in red sticks the original residues and the distances between those residues. (**B**) Number of individuals with CF listed in CFTR2 that carry the variants under study according to the most recent update. “na”—variant not listed in CFTR2.

As missense variants tend to cluster in structural regions that appear particularly vulnerable to mutations (frequently termed hot-spot regions), we focused on variants that are relatively close among themselves. We selected 9 variants of TMD1—R75G, H139R, I148T, D192G, G194R, H199Y, V201M, W361R (T > A) and W361R (T > C)—with an average distance of 12 Å and 5 variants of NBD1—L558S, A559T, A559E, R560T and L571S—with an average distance of 8 Å (Figure 2A and Figure S1). All variants analyzed are rare, although with variable incidence (Figure 2B). Detailed information about the mutations analyzed is included as Supplemental Information.

### 2.2. Impact of Mutations upon CFTR Processing and Response to Correctors

**Figure 3:**
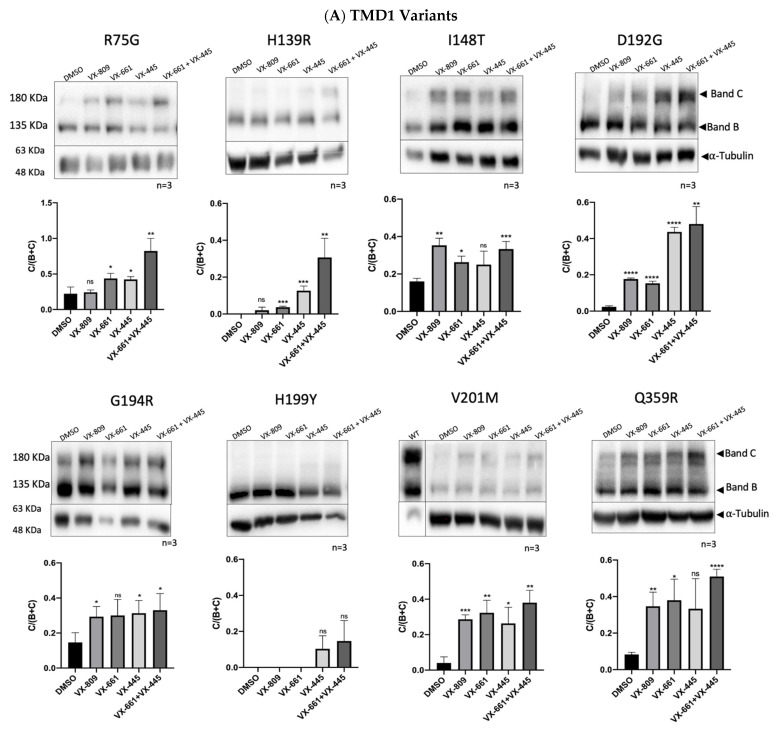

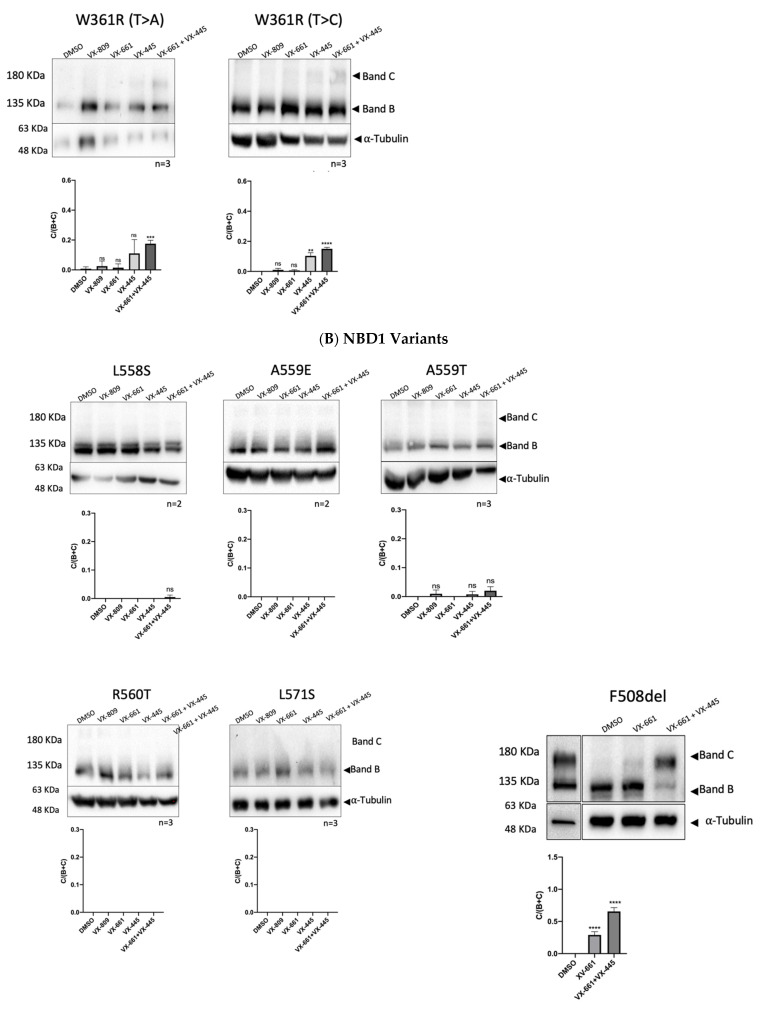

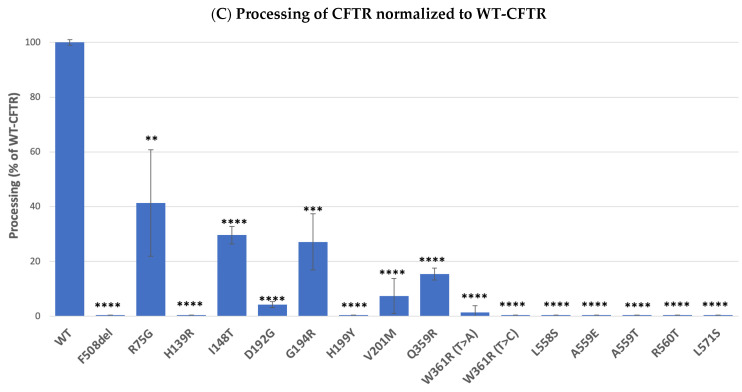
WB analysis of CFBE cells expressing CFTR variants after incubation with 0.1% DMSO (*v*/*v*), VX-809 (3 µM), VX-661 (5 µM) alone or in combination with VX-445 (3 µM) for 48 h. (**A**) TMD1 variants R75G-, H139R-, I148T-, D192G-, G194R-, H199Y, V201M-, Q359R-, W361R (T > A), W361R (T > C)-CFTR. (**B**) NBD1 variants L558S-, A559E-, A559T-, R560T- and L571S-CFTR. WT-CFTR control is shown close to V201M-CFTR. For each condition, densitometry was used to calculate the percentage of mature CFTR (Band C/Band B + Band C). Data are shown as mean ± SEM. Asterisks indicate significant differences compared with DMSO (*p*-value < 0.05, unpaired *t*-test). Images were acquired using ChemiDoc XRS+ imaging system BIO-RAD and further processed by Image Lab 6.0.1 software. (**C**) Processing of CFTR variants normalized to WT-CFTR. Asterisks indicate significant differences compared to WT-CFTR. * *p*-value < 0.05; ** *p*-value < 0.01; *** *p*-value < 0.001; **** *p*-value < 0.0001 (two-tailed unpaired Student’s *t*-test).

We assessed the impact of the variants on the expression and processing of CFTR and the efficacy of different small molecule compounds in the rescue of the CFTR mutants in the study. To accomplish this, mutations were introduced into full-length CFTR cDNA, which was used to stably transduce CFBE cells, and CFTR maturation was monitored by WB (Figure 3). Stably transduced CFBE cell lines were treated for 48 h with the following compounds: 3 µM VX-809/lumacaftor, 5 µM VX-661/tezacaftor, 3 µM VX-445/elexacaftor, with the combination of 5 µM VX-661 + 3 µM VX-445 and DMSO 0.1% (*v*/*v*) as a vehicle control. Total protein was extracted, and WB was used to assess the maturation status/rescue of CFTR containing these variants (Figure 3). CFTR trafficking was evaluated by the glycosylation status of two natural N-linked glycosylation sites in TMD2 which are core glycosylated in the ER (Band B) and then modified by complex glycosylation in the Golgi (Band C).

Under control conditions, only the immature (Band B) form of CFTR was detected for most CFTR mutants, indicating that traffic through the Golgi is impaired and thus confirming them as Class II mutations. The exceptions are R75G, I148T, D192G, G194R, and Q359R, which led to a significant reduction of CFTR processing compared to WT-CFTR despite not abrogating completely the appearance of Band C (Figure 3C). For NBD1 variants, no band C is observed.

R75G is the variant that shows the highest processing under control conditions and after treatment with modulators. Under control conditions, its processing efficiency is 41%, which is significantly increased by modulator treatment, except for VX-809. The combination of VX-661 and VX-445 results in processing levels close to WT-CFTR.

H139R abolishes the processing of CFTR, VX-661, VX-445, and the combination of both significantly increased the amount of mature protein. In contrast, VX-809 causes only a modest, non-significant, increase in mature CFTR, which is in agreement with data [18] showing that H139R does not respond to VX-809. However, previous studies in HEK cells have shown that H139R-CFTR is processed and that its processing increases after VX-809 treatment [19]—a discrepancy that highlights how the cell background affects the response to modulators.

Although listed in CFTR2 as non-CF causing (but anyway listed as eligible for Trikafta), I148T in CFBE cells impaired CFTR processing (to 30% of WT-CFTR). We also observed that significant amounts of band C could be rescued when the modulators were applied.

For D192G, we detected a residual amount of Band C, that could be significantly recovered by all modulators tested. The impact upon processing is in agreement with previous data on transient expression in COS-1 cells, which reported only a residual amount of band C and a lack of response to low-temperature treatment [20].

**Figure 4:**
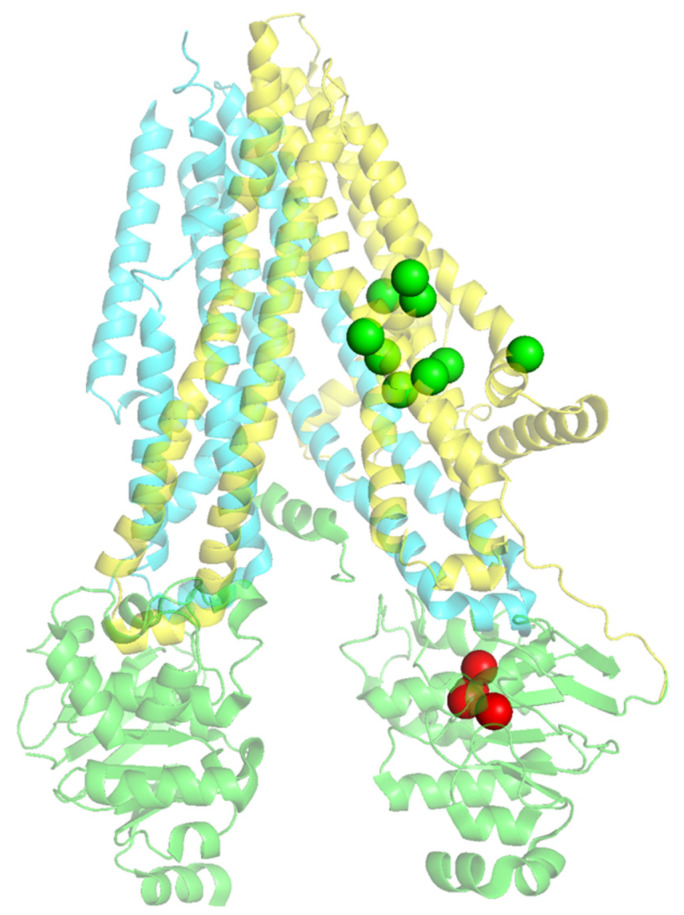
CFTR structure (PDB: 5UAK) with TMD1 in yellow, TMD2 in blue, and NDB1 and NBD2 in green. The green spheres represent the position of the mutations that respond to modulators, R75G, H139R, I148T, D192G, G194R, H199Y, V201M, Q359R, and W361R. The red spheres represent the position of mutations that do not respond to modulators, A559T, A559E, R560T, L558S, and L571S placed at NBD1.

To the best of our knowledge, we provide here the first biochemical characterization of G194R. This variant decreases, but does not abolish, the production of mature CFTR and can be rescued by the tested modulators (Figure 4).

H199Y abolishes the production of band C, which can only be rescued by VX-445 and the combination of VX-661 and VX-445. Recently, a binding pocket for VX-809 and VX-661 was identified in the CFTR structure (6msm; ATP-bound phosphorylated channel) through blind docking [21] and more recently also using cryoEM [22]. H199 is one of the residues that form that pocket, and when mutated to H199A and H199W, a decrease in maturation is observed. This decrease can be fully rescued by VX-809 for the H199A but only partially for H199W, suggesting that this amino acid is relevant in VX-809 induced correction [21].

V201M is classified in CFTR2 as a variant of unknown significance. To the best of our knowledge, we provide here the first molecular characterization of this variant. Under control conditions, V201M decreases (to 7% of WT-CFTR), but does not totally abolish the maturation of CFTR, and this defect can be significantly rescued by almost all modulators. V201M is currently eligible for Trikafta and Symdeco.

Q359R-CFTR decreases, but does not abolish, CFTR processing under control conditions (15% of WT-CFTR) and exhibits a significant response for VX-809, VX-661, and the combination of VX-661 + VX-445.

We studied two versions of the missense mutation W361R: the transversion c.1081T > A and the transition c.1081T > C. We observed that both W361R c.1081T > A and c.1081T > C abolish the appearance of band C CFTR, which is in agreement with a previous study that reports that W361R (c.1081T > A) expressed in HEK293, BHK, and HeLa cells, impairs CFTR processing [23]. The incubation of W361R with the modulators led to a significant increase in CFTR maturation when the combination of VX-661 and VX-445 was applied. The other modulators have a smaller (and in general non-significant) effect. The amounts of band C produced with the modulator VX-809 did not correlate with the maturation level reported previously [23] but the difference may be explained by the much higher VX-809 concentration and the different cell type (10 µM VX-809, HEK293 cells in a previous study versus 3 µM VX-809, CFBE cells here) which can contribute to the different findings.

For L558S stably expressed in CFBE cells, we show that band C is not present under control conditions, which is in agreement with previous reports in HEK293 cells [24]. None of the modulators tested could rescue L558S-CFTR.

In CFMDB, there are three variants reported in residue A559: A559E, A559T, and A559V. We characterized A559E and A559T, which were stably expressed in CFBE cells. A559T is the only variant listed in CFTR2 and is more well documented compared to the other two. No band C was observed for A559T under control conditions and no response to any of the modulators tested was observed, in agreement with previous reports in COS-7 cells [25], and in FRT cells, where only residual levels of band C could be found [26]. A559E is not registered in CFTR2, but a previous study reported a complete abrogation of processing in COS-7 cells [27]. Here, we observe that no Band C is present under control conditions and that none of the modulators tested rescued this effect.

Regarding R560T, no band C could be detected under control conditions and none of the modulators used was able to rescue it.

Like the other NBD1 mutants studied, L571S also abolishes band C production and does not respond to any of the modulators.

Overall, the results in Figure 3 show that all mutants localized in TMD1 respond to modulators—in particular to the combination of VX-661 + VX-445—while none of the NBD1 mutations could be rescued by drugs.

### 2.3. Structural Mapping of Mutations

The data obtained assessing the maturation status of CFTR bearing these variants upon treatment with modulators (Figure 3), allowed us to classify the variants as responders or non-responders to modulators and to map them accordingly in CFTR structure (Figure 4). This graphical representation highlights well-defined zones in the CFTR 3D structure protein with responders mapping to TMD1 and non-responders to NBD1.

**Figure 5:**
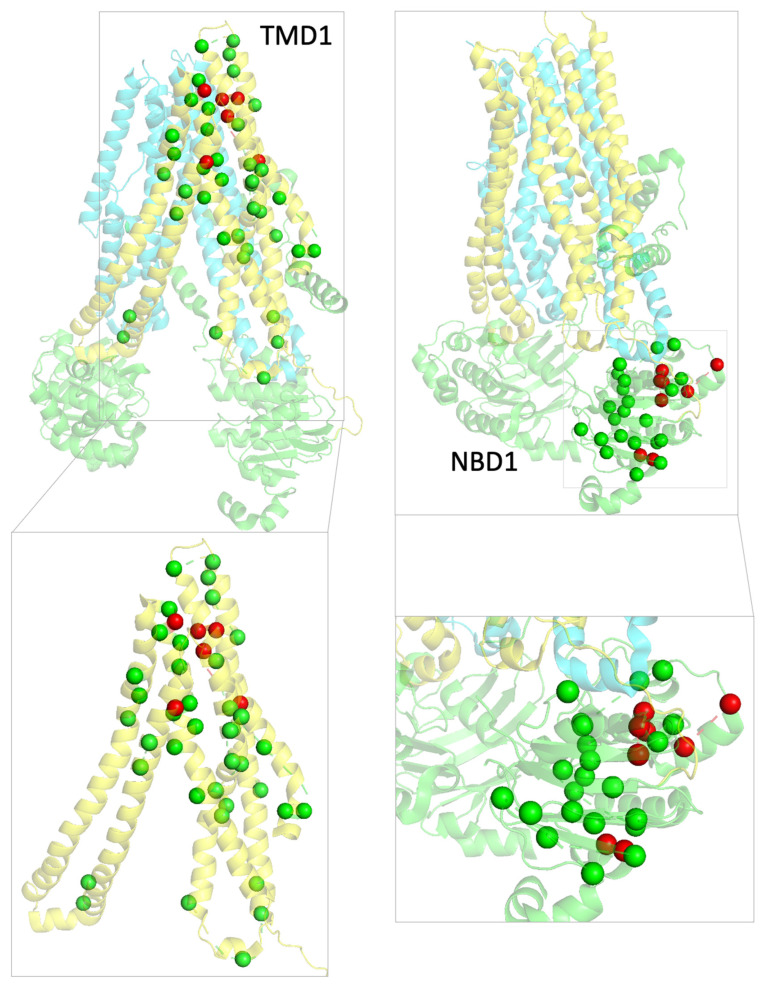
General view of CFTR structure (PDB: 5UAK) with TMD1 in yellow, TMD2 in blue, NDB1 and NBD2 in green. Mutations listed in CFTR2 located in TMD1 or NBD1 are identified as green or red spheres according to their eligibility for Trikafta (green—eligible, red—non eligible). Insets allow a closer view of the distribution of mutations in the two domains.

Considering that 168 missense mutations are eligible for treatment with Trikafta—61 in TMD1, 32 in NBD1, 41 in TMD2, and 18 mutations in NBD2 (Supplementary Table S1), we mapped on CFTR structure all the missense mutations listed in CFTR2 classified as responders (green spheres) or non-responders (red spheres) according to their eligibility for Trikafta (Figure 5 and Supplementary Table S2) to determine if the structural pattern in Figure 4 (for the mutations we studied) could be part of a more extended pattern of response.

The results show that, although the pattern is not as clear as in the smaller number of mutations in our study (Figure 4), most mutations are responsive to the modulators and that lack of response seems to be associated with specific locations. For the TMD1 mutations listed in CFTR2, G91R, P99L, L102R, L227R, R334W are the only ones not eligible for Trkafta, whereas for NBD1, M470V, S492F, I502T, D513G, L558S, A559T, R560K/T/S, A561E, Y563D, Y569D, G576A, H609R, A613T are the mutations listed in CFTR2 that are not eligible for Trikafta (see also Supplementary Table S1).

### 2.4. Impact of the Mutations upon Stability of CFTR Structure

**Figure 6:**
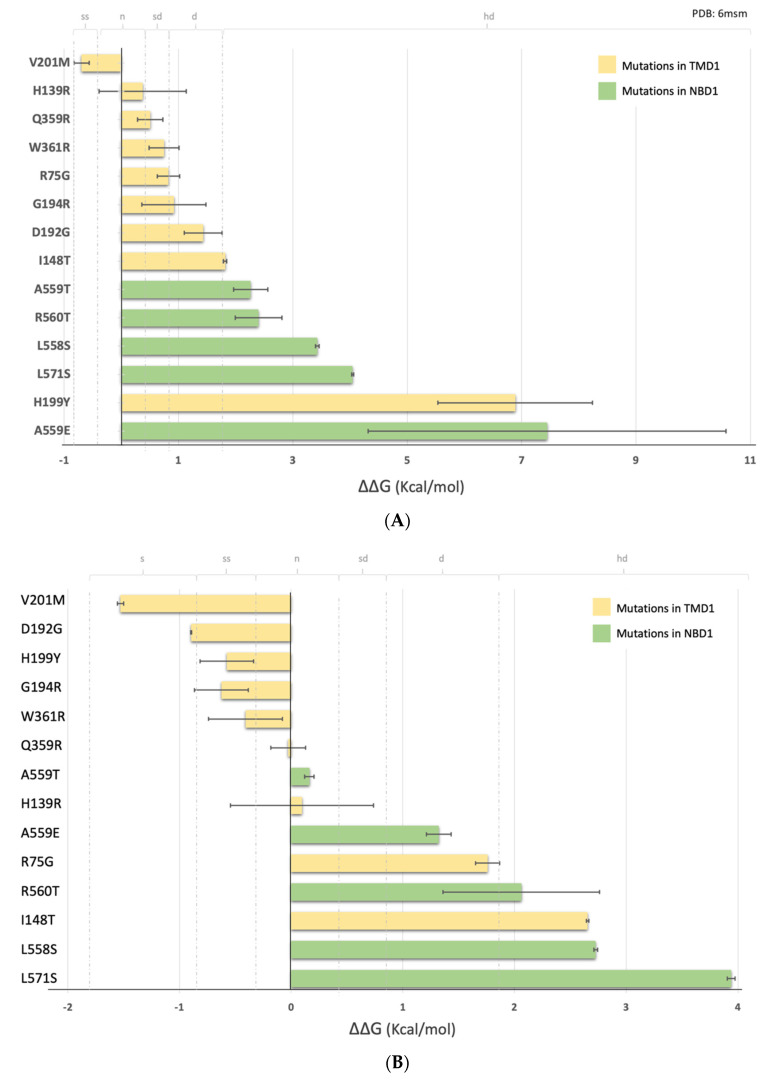

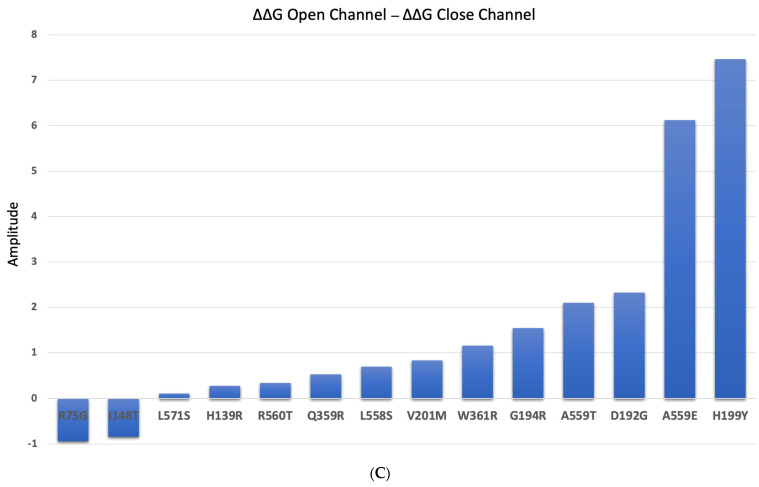
Energy prediction of ΔΔG (kcal/mol) of CFTR mutants calculated with FoldX [29]. (**A**)—using as template the phosphorylated ATP-bound human CFTR (PDB: 6msm)—“open” conformation [28]; (**B**)—using as template the dephosphorylated ATP-free human CFTR (PDB: 5uak)—“closed” channel conformation [7]. The yellow bars correspond to the TMD1 variants, and the green bars correspond to the NBD1 variants. The limits for mutations to be classified as slightly stabilizing (ss), neutral (n), slightly destabilizing (sd), and destabilizing (d) are indicated as dotted gray lines. (**C**)—Variation of ΔΔG (kcal/mol) for CFTR variants calculated as the difference between the ΔΔG “open” and the ΔΔG “closed”. Positive values indicate mutations that are less destabilizing in the closed conformation. Mutations near 0 indicate that the destabilizing effects caused by mutations are almost similar in open or closed conformations. Mutations with negative values are less destabilizing in the open conformation.

To better understand the pattern of response to the modulators, we predicted the effect of these 14 mutations (note that above we analyzed 15 variants, but two of them lead to the same amino acid substitution) on CFTR stability. The data were analyzed in terms of predicted free energy differences (ΔΔG values) of each CFTR mutant, when compared to the WT, using two of the available structures: one corresponding to the ATP-bound phosphorylated conformation (pbd:6msm) [28] (called “open” for easier comparison) and one corresponding to the ATP-free dephosphorylated conformation (pdb:5uak) [7] (called “closed” throughout) (Figure 6 and Table 1). To perform the calculations, we used FoldX [29]. According to a recent report [30], FoldX outperformed the other algorithms due to its success in correctly predicting the effects of mutations on the stability of CFTR NBDs. In this program the effect of mutations can be categorized according to their effect on the conformational energy (the more negative the more stable, the more positive the more unstable): highly stabilizing (ΔΔG < −1.84 kcal/mol); stabilizing (−1.84 kcal/mol ≤ ΔΔG < −0.92 kcal/mol); slightly stabilizing (−0.92 kcal/mol ≤ ΔΔG < −0.46 kcal/mol); neutral (−0.46 kcal/mol < ΔΔG ≤ +0.46 kcal/mol); slightly destabilizing (+0.46 kcal/mol < ΔΔG ≤ +0.92 kcal/mol); destabilizing (+0.92 kcal/mol < ΔΔG ≤ +1.84 kcal/mol); highly destabilizing (ΔΔG > +1.84 kcal/mol).

Besides the 14 missense mutations under study, we included in the ΔΔG energy prediction calculation the F508del, as it is the most common CF-causing mutation. As we can see in Tab 1, the predicted energies for this mutant are high in relation to other mutants, which can be mostly due to the distinct impact of a deletion when compared to a simple amino acid substitution upon the algorithm predictions.

Table 1 shows the calculated ΔΔG (kcal/mol) for the 14 mutants under analysis (9 in TMD1 and 5 in NBD1) plus F508del, using the two CFTR structures (“open” [28] and closed [7]) as templates.

In both conformations, we observed that TMD1 mutations are less destabilizing (less positive ΔΔG values) than NBD1 mutations (more positive ΔΔG values) (Figure 6A,B). In the open conformation, the average ΔΔG value for mutations in TMD1 is 1.42 kcal/mol (or 0.74 kcal/mol if H199Y is excluded) while for NBD1 it is 3.92 kcal/mol (Supplementary Table S3). A Welch two-sample *t*-test showed that this difference is statistically significant (t(5) = 2.570, *p* = 0.022) if H199Y is excluded from the calculation. For the closed conformation, the average ΔΔG is 0.05 kcal/mol for TMD1 and 2.04 kcal/mol for NBD1 (Supplementary Table S3)—a difference that is also statistically significant (t(8) = 2.306, *p* = 0.033). According to our calculations, V201M-CFTR is the only mutation predicted to stabilize CFTR (negative ΔΔG values) both in the open and closed conformations (−0.693 kcal/mol and −1.527 kcal/mol, respectively). Comparing the open or closed conformations, we observed that predicted ΔΔG values for some mutants can be very different—as is the case of H199Y, which is predicted to be highly destabilizing in the open conformation (6.88 kcal/mol) and slightly stabilizing in the closed conformation (−0.57 kcal/mol) (Table 1). These differences are shown in Figure 6C. Most of the mutations analyzed are more destabilizing when CFTR is in the ‘open’ conformation. The only mutations that are shown to be more stable when CFTR is in its “open” state are I148T and R75G which correspond to those with higher levels of band C under control conditions (Figure 3C).

In general, for both conformations (open and closed), TMD1 variants are predicted to cause smaller destabilizing effects than the selected NBD1 variants.

### 2.5. Impact of the Mutations upon Stability of CFTR Structure in the Presence of Modulators

Next, we used the structures of phosphorylated human CFTR in the presence of Mg/ATP and either VX-809 (pdb:7svd) or VX-661 (pdb:7sv7) [22] as templates to predict ΔΔG changes (Kcal/mol) induced by the mutations using FoldX [29].

**Figure 7:**
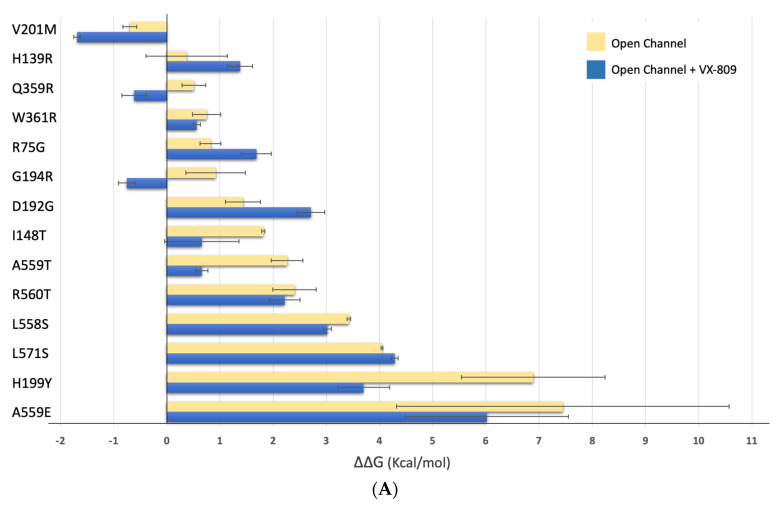

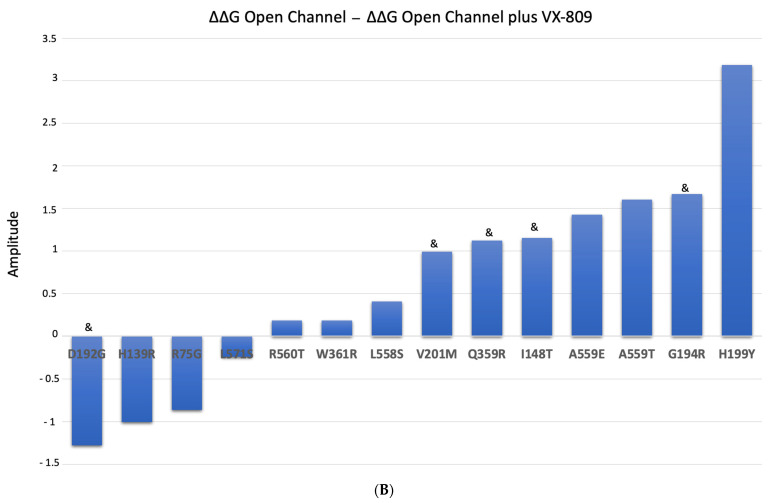
Energy prediction of ΔΔG (kcal/mol) of CFTR mutants in the presence and absence of VX-809 calculated with FoldX [29]. (**A**)—yellow bars: human CFTR bound to phosphorylated ATP (6msm)—“open” conformation [28], (similar to Figure 6A); blue bars: human CFTR + Mg/ATP + VX-809 (pdb:7svd) [22]; bars are presented as mean ± standard deviation. (**B**)—Variation of ΔΔG (kcal/mol) for CFTR variants calculated as the difference between the ΔΔG “open” and the ΔΔG “open” in the presence of VX-809. “&” corresponds to variants in which band C is present.

Figure 7A shows that, in general, there is a gain in predicted stability for most mutants in the presence of VX-809 (V201M, Q359R, W361R, G194R, I148T, A559T, R560T, L558S, H199Y and A559E). The exception is observed for mutations H139R, R75G, D192G, and L571S, where the presence of the corrector appears to negatively impact CFTR stability (Figure 7B). When we consider the effect of this modulator by domain, we observe a decrease from 1.42 kcal/mol to 0.85 kcal/mol for mutations in TMD1, which corresponds to a general gain in stability (Suppl Table S3). Regarding NBD1, the change in predicted ΔΔG energies is almost negligible—from 3.92 kcal/mol to 3.24 kcal/mol (Supplementary Table S3).

In Figure 3A, we have shown that VX-809 treatment significantly rescues CFTR in mutants I148T, D192G, G194R, V201M and Q359R. Calculating the average of the predicted ΔΔG energies for mutations that respond or do not respond to VX-809, we determined a value of 0.06 kcal/mol for mutations that are rescued by VX-809 and 2.61 kcal/mol for those that are not (Supplementary Table S3). These observations indicate that mutations that respond to VX-809 have a minimal impact upon CFTR stability, a statistically significant finding (t(9) = 2.26, *p* = 0.027).

**Figure 8:**
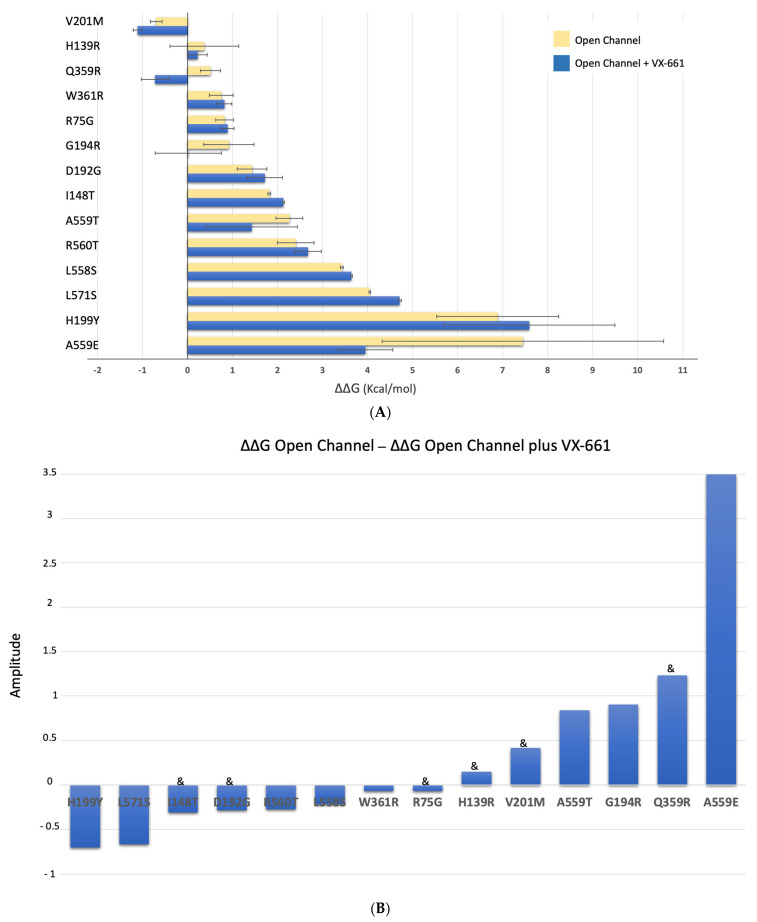
Energy prediction of ΔΔG (kcal/mol) of CFTR mutants in the presence and absence of VX-661 calculated with FoldX [29]. (**A**)—yellow bars: human CFTR bound to phosphorylated ATP (6msm)—“open” conformation [28] (similar to Figure 6A); blue bars: human CFTR + Mg/ATP + VX-661 (pdb:7sv7) [22]; bars are presented as mean *±* standard deviation. (**B**)—Variation of ΔΔG (kcal/mol) for CFTR variants calculated as the difference between the ΔΔG “open” and the ΔΔG “open” in the presence of VX-661. “&” corresponds to variants in which band C is present.

Figure 8A shows that, overall, there is a gain in predicted energy stability in the presence of VX-661 for V201M, H139R, Q359R, G194R, A559T and A559E and a loss of predicted stability for W361R, R75G, D192G, I148T, R560T, L558S, L571S and H199Y—as shown in the changes represented in Figure 8B. For TMD1 mutations, predicted ΔΔG energies have a small decrease from 1.42 kcal/mol to 1.28 kcal/mol, which means a minor gain in stability (Supplementary Table S3). NBD1 mutations have similar behavior, since predicted ΔΔG energies decrease from 3.92 kcal/mol to 3.28 kcal/mol (Supplementary Table S3).

As above for VX-809, we also observed that the average of ΔΔG predicted energies is 0.5 kcal/mol for mutants in which a significant amount of band C was observed by WB and 3.1 kcal/mol for mutants in which no band C was present. Therefore, as previously reported for VX-809, mutations that respond to VX-661 seem to cause less impact on CFTR stability compared to those that do not respond, an observation that is statistically significant (t(11) = 2.20, *p* = 0.026).

## 3. Discussion

Considering that missense mutations tend to cluster around hot spots, here we analyzed two groups of CFTR mutations in TMD1 and NBD1 that are relatively close (average of 12 Å for TMD1 mutations and 8 Å for NBD1 mutations) to each other. These two CFTR domains concentrate about 40% of all CFTR missense mutations, associated with a diversity of effects upon protein maturation [31]. In CFTR, several hotspots have been reported [21,32]: e.g., in TMDs, formed by residues lining the pore (G85E, E92K, D110H, P205S, R334W, I336K, T338I, S341P, R347H/ R347P, R352Q); in residues involved in the assembly of the ICL bundle (G178E/G178R, G970R); at the NBD1:ICL4 interface (S492F, I507del, F508del, V520F, A559T, R560K/R560T, A561E, H1054D, G2061R, L1065P, R1066H/R1066C, F1074L, L1077P); or at the canonical ATP-binding site (S549N, S549R, G551D/G551S, G1244E, S1251N, S1255P) [32]. We studied a set of 14/15 CFTR mutations located in either TMD1 or NBD1 for their effect upon CFTR processing, their response to the double corrector combination VX-661/VX-445 and then correlated the results with the impact of each mutation on CFTR overall stability as predicted by FoldX. For this set of mutations, we show that the TMD1 mutations respond to the corrector combination and have a modest effect upon stability whereas NBD1 mutants do not respond and have a strong destabilizing effect upon CFTR. Although focusing on CFTR expression /processing (and not channel function), this observed correlation suggests that assessing CFTR conformational stability by these methods may provide information, at least for certain specific locations within the protein, on the possible response of mutant protein to modulators.

**Figure 9:**
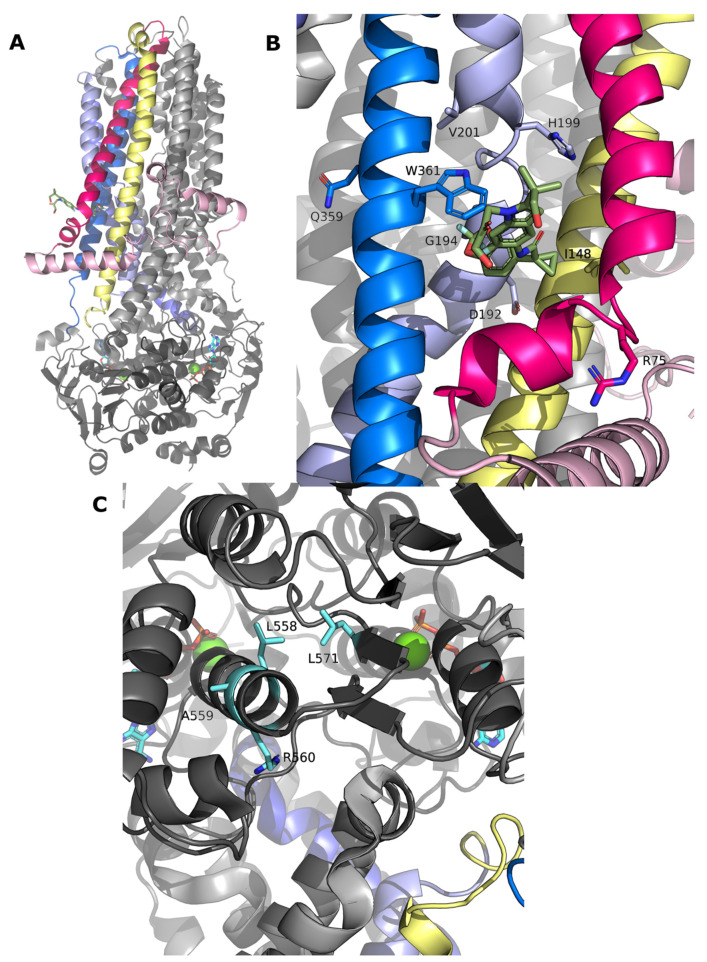
(**A**) Overall CFTR structure in the presence of VX-661 (PDB:7SV7), colored in green sticks. (**B**) Zoom of the binding region of VX-661 at CFTR structure. Residues analyzed in this work are colored and labeled in sticks. (**C**) Zoom of the NBD1 region, where some residues analyzed are located. Side chains of the residues are labeled and represented as cyan sticks.

R75G leads to a decrease in CFTR processing (41% of WT) and responds to almost all the modulators tested. The fact that, except for VX-809, all modulators significantly increase the amount of band C could indicate that the binding site of VX-809 is destabilized by this alteration. The proximity of this residue to the binding site [21] of VX-809, and the impact of substituting an arginine by a glycine, may most probably negatively impact the stability of the helix where this residue stands, and consequently modify the shape and properties of the aforementioned VX-809 binding pocket. It is interesting that despite the binding site on CFTR of both VX-809 and VX-661 being the same (see Figure 9), the chemical differences between these two compounds are responsible for the distinct response observed for R75 (Figure 9).

We demonstrate that H139R abolishes CFTR processing, but treatment with VX-661, VX-445, and particularly the combination of both, leads to a significant increase in band C. Although this mutation is located far from the reported binding site of VX-661 (at the TM2 of TMD1—Figure 9B), it is found to be in close proximity with the binding site reported for VX-445 [33].The substitution of a polar amino acid, by a large and positively charged residue such as arginine, can change the arrangement of the helix and negatively impact the interaction with TMD2 (due to its proximity) and the stability of the entire helix, part of which is directly adjacent to the binding site of VX-661 [33].

I148 is directly involved in the binding site of VX-809 and VX-661 (Figure 9B). Replacing this hydrophobic residue by the polar threonine can lead to destabilization of TMD1. Interaction with the correctors leads to increased stabilization of this region of the protein, which ultimately is translated into the overall stabilization of CFTR and the observed appearance of band C for I148T after modulator treatment.

According to the available structures of CFTR in the presence of VX-661 and VX-809, D192 is close to the compound binding sites (Figure 9B). Under control conditions, we observed that a small amount of band C is present in D192G (4% of WT levels) and that all modulators significantly rescued CFTR with this variant, since they probably have a countereffect on its destabilization effect.

Under control conditions, G194R decreases processing to 27% of WT-CFTR, which is increased by all modulators tested. Most likely the substitution of glycine by positively charged arginine changes the original kink, which is near the binding region of both VX-661 and VX-809. In their presence, the destabilizing effect caused by this mutation is reverted, most probably due to their stabilizing effect on this region of the protein (Figure 9B).

We provide here the first molecular characterization for H199Y; of all the TMD1 variants analyzed it is the one with the most modest response to modulators, which is similar to the response also observed elsewhere for G85E [34] (Figure 9B) and in agreement with the fact that the two residues are close to each other in CFTR structure. Interestingly, this is the TMD1 mutation that has the strongest destabilizing effect as assessed by FoldX. Interestingly also, it was also the one with the greatest change when comparing the impact upon the unphosphorylated and the phosphorylated CFTR structure—suggesting that it is located at a very flexible spot.

Our results show that V201M severely affects CFTR processing but that this defect can be rescued by all the modulators tested. The side chain of this residue is found to be at the surface of CFTR, close to the binding region of both VX-661 and VX-809, again explaining the stabilizing effect observed in our results (Figure 9B).

Under control conditions, Q359R-CFTR has 15% of WT-CFTR processing and is particularly responsive to the VX-661 and VX-445 combination. Q359 is also near the reported binding region of both VX-661 and VX-809, possibly explaining their stabilizing effect on CFTR structure (Figure 9B).

W361 was recently identified as a major player in the binding of VX-809/VX-661 to CFTR, establishing tight contacts with the benzodioxolyl core (common molecular part of both modulators) (Figure 9B). The modest, albeit significant, response after treatment on W361R mutant probably reflects this residue’s critical role in accommodating correctors such as VX-809/VX-661.

For the 4 NBD1 mutations tested, we obtained similar results. None of the modulators tested was able to rescue them. The positions where the tested modulators bind are considerably distant from the region where these mutations are found and, although modulators are able to induce an allosteric effect that leads to correction of NBD1 mutations, particularly F508del, such an effect is most likely not enough to overcome the defect caused by these mutations (Figure 9C).

As stated above, using a Welch’s *t*-test to analyze the statistical relevance of the differences obtained by the predicted energies of ΔΔG by FoldX, we observed that these TMD1 mutations—that we observed as easier to correct—are always predicted to have a smaller destabilizing effect towards CFTR than NBD1 mutations. This may be explained by the fact that the reported binding regions for CFTR modulators are located in CFTR TMDs, in close proximity with several missense mutations evaluated in this work. The combination between the effect on the stability of the protein caused by the mutations, together with the fact that most of the evaluated modulators bind and stabilize the TMDs, indicates that with the currently available modulators, it is easier to correct mutations found in this domain of CFTR when compared to mutations found in NBD1, which can possibly be corrected when the allosteric effect caused by modulator binding is enough to overcome a distant structural alteration.

Prediction of the effect of the mutations upon global CFTR is a difficult task. What we present here does not configure a global approach but highlights two specific regions in TMD1 and NBD1 which seem to be in one case prone to correction and in the other refractory to the tested correctors. Additionally, we performed a comparative analysis using the list of mutations in NBD1 and TMD1 eligible for Trikafta (Supplementary Table S2) and assessing their impact upon CFTR structure using FoldX (Supplementary Table S4). This is a “biased” analysis, as, for this list, the “starting data” is that these mutations have been included in a label extension (thus lacking any reports on level of correction or any other parameter that can help distinguish the mutations). Interestingly, however, there is no statistical difference between the stability of the TMD1 mutants versus the NBD1 ones whereas for the restricted number of mutations analyzed in our study the values are statistically different (for the 6msm structure).

Our analysis here was restricted to FoldX, based mainly on recent data showing that it outperforms other algorithms but also that it is one of the most accurate programs for predicting the effect of mutations in membrane proteins [35]—despite its limitations of being developed based mostly on destabilizing mutations and of not being very accurate in reflecting long-range allosteric interactions. Despite these aspects, our results agree with recent studies using spatial covariance analysis—which show that the impact of the residues assessed in our work upon CFTR trafficking efficiency is significantly higher for NBD1 residues than for TMD1 ones [36,37].

In conclusion, here we identified two structural locations in CFTR that associate respectively with response or absence of response to the double corrector combination VX-661/VX-445 and we found that this distinction correlates with the impact of mutations on CFTR stability (as predicted by FoldX). Our study highlights the ability of in silico predictions to help understanding response to modulators while still showing that such observations/predictions cannot be expanded to full-length CFTR without caution.

## 4. Methods and Materials

### 4.1. Site-Directed Mutagenesis

The cDNA of human wild-type CFTR (wt-CFTR) cloned in the pcDNA™5/FRT vector (an 8.1 kb vector containing an ampicillin resistance gene) was used as the template for the introduction of each mutation by site-directed mutagenesis using KOD HOT start DNA polymerase (Novagen, Darmstadt, Germany), with primers listed in Table 2. The sequence and the insertion of the mutations were analyzed by the Sanger sequencing.

### 4.2. Cell Lines

Novel cell lines were generated by lentiviral transduction of the CFBE41o- cell line [38]. After site-directed mutagenesis, CFTR cDNA with the variant of interest was re-cloned into the lentiviral expression vector pLVX-Puro that was transfected into the packaging cell line 293T for the production of lentiviral particles. These particles were used to transduce parental CFBE41o- cells. After selection with puromycin 5 mg/mL for 15 days, the efficiency of the transduction was assessed by WB to confirm CFTR expression [39,40].

### 4.3. Treatment with CFTR Modulators

CFBE cells were seeded in a 24-well plate and incubated with either 3 μM of the corrector VX-809/Lumacaftor (Selleckchem, Houston, TX, USA) [15], 5 μM of the corrector VX-661/Tezacaftor) (Selleckchem, USA), 3 μM of the next-generation corrector VX-445/Elexacaftor [41], the combination of 5 μM VX-661/3 μM VX-445, or DMSO 0.1% (*v*/*v*) as vehicle control. All modulators were diluted in medium supplemented with 0.1% (*v*/*v* FBS). Protein extracts were prepared after 48 h incubation with modulators.

### 4.4. Western Blot Analysis

Equal volumes of protein extracts prepared from equivalent number of cells were loaded onto SDS-PAGE gels to analyze CFTR expression and processing. Gels were then transferred to polyvinylidene difluoride (PVDF) membranes (Millipore, Burlington, MA, USA). The primary antibody was the anti-CFTR monoclonal antibody 596 (CFF) (against amino acids 1204-1211) at 1:3000 dilution and the secondary antibody was horseradish peroxidase-labeled anti-mouse IgG at 1:3000 (BioRad, Hercules, CA, USA) [39]. Alpha tubulin was used as loading control and detected by anti-alpha tubulin antibody (1:10,000) (Sigma, Darmstadt, Germany). Images were acquired using ChemiDoc XRS+ imaging system BioRad and analyzed with the Image Lab 6.0.1 software.

### 4.5. Statistical Analyses

Data are mean values ± SEM. Statistical analyses were performed on GraphPad Prism 7.0 using two-tailed unpaired Student’s *t*-tests with *p* < 0.05 considered significant. A Welch’s *t*-test, using Microsoft Excel was also performed when samples to compare do not have the same size.

### 4.6. Protein Structure Preparation

Several full-length structures were downloaded from the PDB [42], corresponding to CFTR structures in both “open” (phosphorylated, ATP-bound) and “closed” (dephosphorylated, ATP-free) states, and in the presence and absence of chemical modulators. The structure with pdb code 5uak [7] stands for dephosphorylated ATP-free CFTR in its closed state in the absence of modulators; the structure with reference 7svr [22] represents CFTR in its dephosphorylated closed state in the presence of chemical modulator Lumacaftor (VX809); 6msm [28] represents the human CFTR protein structure in its phosphorylated open conformation with ATP bound and in the absence of modulators; and finally 7sv7 and 7svd [22] represent different structures of the human CFTR protein in its open phosphorylated state with Mg and ATP bound molecules, in the presence of Tezacaftor (VX661) and Lumacafor (VX809) modulators, respectively.

Each structure was prepared by removing all water molecules and adding explicit hydrogens according to the protonation state of the titrable amino acid side-chains at pH 7. To perform such calculations, the PypKA program developed by Reis et al. [43] was used. When available, the Mg and ATP molecules were kept for the ΔΔG calculations.

### 4.7. ΔΔG Calculations of CFTR Mutants Using FoldX

To predict the ΔΔG values for the different mutants (R75G, H139R, I148T, D192G, G194R, H199Y, V201M, Q359R, W361R, L558S, A559T, A559E, R560T, and L571S) on the different input CFTR structures previously described, the FoldX force field based-algorithm was used [29]. The implemented method calculates the differences in energy of unfolding (Δ*G*) between the WT structure and a specific mutation based on the sum of different energy terms weighted by empirical factors which characterizes protein residue interactions (e.g., van der Waals interactions, hydrogen bonding, electrostatics, solvation effects, and entropy)—(Equation (1)).

(1) $$\begin{array}{l} \Delta G\left(\mathrm{kcal}/\mathrm{mol}\right)=W_{\mathrm{vdw}}\Delta G_{\mathrm{vdw}}+W_{\mathrm{solvH}}\Delta G_{\mathrm{solvH}}\;\\ +W_{\mathrm{solvP}}\Delta G_{\mathrm{solvP}}+\Delta G_{\mathrm{wb}}+\Delta G_{\mathrm{hbond}}\;\\ +\Delta G_{\mathrm{el}}+\Delta G_{\mathrm{Kon}}+W_{\mathrm{mc}}T\Delta S_{\mathrm{mc}}\\ +W_{\mathrm{sc}}T\Delta S_{\mathrm{sc}} \end{array}$$

To calculate the ΔΔ*G* values, each input structure (the different multiple WT CFTR used structures, previously prepared) is corrected using the RepairPDB function from FoldX, which based on the calculated total energy, corrects problematic torsional angles and atomic clashes. To model each mutation, the FoldX “BuildModel” function was used to generate 100 models for the WT protein (to increase the statistical significance of the results). Afterward, each of the 100 models is mutated by selecting a random rotamer for the mutated residue. In the end, the energies of all WT structures and mutant generated models are calculated on the optimized structures by using a rotamer library applied solely to the amino acid sidechains. The final ΔΔG, for each mutation, is calculated using the formula ΔΔ*G* = Δ*G*_mutant_ − Δ*G*_wildtype_ as the correspondent Standard deviation derived from the 100 generated models.

An exception to this protocol was applied solely to mutant F508del. In this situation, previously to apply the described FoldX protocol, we built a structural model for F508del, using the model loop routine from software Modeller [44,45]. For each one of the generated models, the only region allowed to change, was the one including the 5 amino acids closer (before and after) to the target deletions. Afterward, to calculate the Δ*G*_mutant_ for this mutation, we used the previously described FoldX protocol, using a dummy mutation, where the first residue found in each PDB structure, was mutated to itself.

## Figures and Tables

**Table 1:** Predicted ΔΔG Values ((ΔG(Mutant) − ΔG(WT)) (kcal/mol) of 14 CFTR Mutations (9 in the TMD1 domain and 5 in the NBD1 domain) using FoldX [29]. For these calculations, the following structures were used as templates: dephosphorylated ATP-free human CFTR (PDB: 5uak) [7]; phosphorylated ATP-bound human CFTR (PDB: 6msm) [28]; phosphorylated human CFTR + Mg/ATP + VX-809 (PDB: 7svd) [22] and phosphorylated human CFTR + Mg/ATP + VX-661 (PDB: 7sv7) [22]. Values of ΔΔG > +1.84 kcal/mol are shown in bold.

		CLOSED	“OPEN”	OPEN + VX-809	OPEN + VX-661
	PDB	5uak	6msm	7svd	7sv7
	Mutation				
**TMD1**	R75G	1.760 ± 0.108	0.824 ± 0.196	1.685 ± 0.281	0.890 ± 0.140
	H139R	0.097 ± 0.641	0.374 ± 0.763	1.379 ± 0.236	0.224 ± 0.212
	I148T	**2.654 ± 0.010**	**1.817 ± 0.029**	0.661 ± 0.699	**2.127 ± 0.020**
	D192G	−0.895 ± 0.003	1.433 ± 0.331	**2.711 ± 0.258**	1.716 ± 0.390
	G194R	−0.624 ± 0.241	0.919 ± 0.560	−0.751 ± 0.155	0.015 ± 0.735
	H199Y	−0.576 ± 0.240	**6.888 ± 1.351**	**3.700 ± 0.489**	**7.591 ± 1.902**
	V201M	−1.527 ± 0.028	−0.693 ± 0.131	−1.687 ± 0.061	−1.109 ± 0.099
	Q359R	−0.026 ± 0.156	0.508 ± 0.223	−0.616 ± 0.230	−0.724 ± 0.303
	W361R	−0.408 ± 0.331	0.749 ± 0.263	0.564 ± 0.067	0.817 ± 0.168
**NBD1**	L558S	**2.726 ± 0.017**	**3.426 ± 0.032**	**3.020 ± 0.073**	**3.641 ± 0.017**
	A559T	0.163 ± 0.041	**2.263 ± 0.299**	0.659 ± 0.118	1.425 ± 1.014
	A559E	1.321 ± 0.111	**7.446 ± 3.128**	**6.019 ± 1.533**	**3.949 ± 0.611**
	R560T	**2.060 ± 0.701**	**2.401 ± 0.408**	**2.216 ± 0.288**	**2.673 ± 0.297**
	L571S	**3.939 ± 0.036**	**4.045 ± 0.018**	**4.285 ± 0.067**	**4.712 ± 0.034**
	F508del	**9.699 ± 0.012**	**13.294 ± 0.576**	**4.401 ± 0.010**	**15.937 ± 0.119**

**Table 2:** List of primers used in site-directed mutagenesis. Highlighted in red the codon for mutation.

Mutation		Sequence (5′-3′)
R75G	For	CATTAATGCCCTTCGGGGATGTTTTTTCTGGAG
	Rev	CTCCAGAAAAAACATCCCCGAAGGGCATTAATG
H139R	For	ACACTGCTCCTACGCCCAGCCATTTTTG
	Rev	CAAAAATGGCTGGGCGTAGGAGCAGTGT
I148T	For	TTTGGCCTTCATCACACTGGAATGCAGATGAGA
	Rev	TCTCATCTGCATTCCAGTGTGATGAAGGCCAAA
D192G	For	AACCTGAACAAATTTGGTGAAGGACTTGCA
	Rev	TGCAAGTCCTTCACCAAATTTGTTCAGGTT
G194R	For	CAAATTTGATGAAAGACTTGCATTGGC
	Rev	GCCAATGCAAGTCTTTCATCAAATTT
H199Y	For	GACTTGCATTGGCATATTTCGTGTGGATCG
	Rev	CGATCCACACGAAATATGCCAATGCAAGTC
V201M	For	TTGGCACATTTCATGTGGATCGCTCCTTTG
	Rev	CAAAGGAGCGATCCACATGAAATGTGCCAA
Q359R	For	CCCTGGGCTGTACGAACATGGTATGACTCTCTT
	Rev	AAGAGAGTCATACCATGTTCGTACAGCCCAGGG
W361R (T- > A)	For	GGGCTGTACAAACAAGGTATGACTCTCTTG
	Rev	CAAGAGAGTCATACCTTGTTTGTACAGCCC
W361R (T- > C)	For	GGGCTGTACAAACACGGTATGACTCTCTTG
	Rev	CAAGAGAGTCATACCGTGTTTGTACAGCCC
L558S	For	CGAGCAAGAATTTCTTCAGCAAGAGCAGTATAC
	Rev	GTATACTGCTCTTGCTGAAGAAATTCTTGCTCG
A559T	For	CGAGCAAGAATTTCTTTAACAAGAGCAGTATACAAAG
	Rev	CTTTGTATACTGCTCTTGTTAAAGAAATTCTTGCTCG
A559E	For	CGAGCAAGAATTTCTTTAGAAAGAGCAGTATAC
	Rev	GTATACTGCTCTTTCTAAAGAAATTCTTGCTCG
R560T	For	GCAAGAATTTCTTTAGCAACAGCAGTATACAAAGATGCTG
	Rev	CAGCATCTTTGTATACTGCTGTTGCTAAAGAAATTCTTGC
L571S	For	GATGCTGATTTGTATTTATCAGACTCTCCTTTTGGATACC
	Rev	GGTATCCAAAAGGAGAGTCTGATAAATACAAATCAGCATC

## Data Availability

Not applicable.

## Supplementary Materials

The following supporting information can be downloaded at: https://www.mdpi.com/article/10.3390/ijms24043211/s1. References [7,8,20,21,24,25,26,32,46,47,48,49,50,51,52,53,54,55,56,57,58,59,60,61,62,63,64,65,66,67,68,69,70,71,72,73,74,75,76] are cited in the supplementary materials.
