# Supplementary material for: Rescue of Rare CFTR Trafficking Mutants Highlights a Structural Location-Dependent Pattern for Correction
Source: Int J Mol Sci. 2023 Feb 6;24(4):3211. doi: 10.3390/ijms24043211 (PMC9961391; doi:10.3390/ijms24043211)
Supplement: Supplementary file 1 [file ijms-24-03211-s001.zip › ijms-2071953-supplementary.pdf]

Supplementary Information

Supp Table S1: List of all TMD1 and NBD1 residues for which there are missense variants reported in CFMDB and CFTR2 and information on their eligibility for FDA-approved modulators. The domain borders were TMD1 – residues 69-390 and NBD1 – residues 391-645 [1].

TMD1 Residue Numbers	CFMDB Missense Mutations	CFTR2 Missense Mutations	Eligible for Trikafta	Eligible for Symdeko	Eligible for Kalydeco
72	A72T<br>A72D				
74	R74W<br>R74Q	R74W<br>-	R74Q<br>-	R74Q<br>-	R74Q<br>-
75	R75Q<br>R75L	R75Q<br>-	R75Q<br>-	R75Q<br>-	-<br>-
79	W79R				
82	M82V				
84	Y84H				
85	G85E<br>G85V	G85E<br>-	G85E<br>-		
87	F87L<br>F87S				
88	L88S				
89	Y89C				
90	L90S				
91	G91R	G91R			
92	E92K<br>E92D	E92K<br>-	E92K<br>-	E92K<br>-	
98	Q98R<br>Q98P	Q98R<br>-	Q98R<br>-	Q98R<br>-	
99	P99L	P99L			
101	L101S				
102	L102R<br>L102P	L102R<br>-			
105	I105N				
107	A107G				
108	S108F				
109	Y109N<br>Y109C	-<br>-	Y109N<br>-	Y109N<br>-	
110	D110Y<br>D110N<br>D110H<br>D110E	-<br>-<br>D110H<br>D110E	-<br>-<br>D110H<br>D110E	-<br>-<br>D110H<br>D110E	-<br>-<br>D110H<br>D110E
111	P111A<br>P111L				
113	N113I				
116	E116K<br>E116Q	E116K<br>-	E116K<br>-	E116K<br>-	
117	R117H<br>R117C	R117H<br>R117C	R117H<br>R117C	R117H<br>R117C	R117H<br>R117C

	R117G<br>R117L<br>R117P	R117G<br>R117L<br>R117P	R117G<br>R117L<br>R117P	R117G<br>R117L<br>R117P	R117G<br>R117L<br>R117P
119	I119V				
120	A120T<br>A120V	A120T<br>-	A120T<br>-	A120T<br>-	A120T<br>-
122	Y122H<br>Y122C				
124	G124R				
125	I125T				
126	G126D	G126D	G126D	G126D	
130	L130V				
131	I132M				
137	L137R<br>L137H<br>L137P				
139	H139R<br>H139L	H139R<br>-	H139R<br>-		
140	P140S<br>P140L				
141	A141D				
145	L145H				
146	H146R				
148	I148T<br>I148N	I148T<br>-	I148T<br>-	I148T<br>-	I148T<br>-
149	G149R<br>G149V				
151	Q151K				
152	M152L<br>M152V<br>M152R	-<br>-<br>-	-<br>M152V<br>-	-<br>M152V<br>-	-<br>M152V<br>-
155	A155P				
158	S158R<br>S158N<br>S158T				
159	L159S				
161	Y161D<br>Y161N<br>Y161S	Y161D<br>-<br>-	-<br>-<br>Y161S		
162	K162E				
163	K163T				
164	T164A				
165	L165S	L165S	L165S		
166	K166Q				
170	R170H<br>R170G<br>R170C	R170H	R170H	R170H	R170H
174	I175V				
177	I177T<br>I177T				
178	G178R<br>G178E	G178R<br>-	G178R<br>-	G178R<br>-	G178R<br>-
179	Q179K				

183	L183I				
186	N186K				
187	N187K				
189	N189S<br>N189K				
191	-	F191V	F191V	F191V	
192	D192G<br>D192N	D192G<br>-	D192G<br>-	D192G<br>-	D192G<br>-
193	E193K	E193K	E193K	E193K	E193K
194	G194V<br>G194R	G194V<br>G194R	G194V<br>G194R	G194V<br>G194R	-<br>G194R
198	A198P<br>A198T				
199	H199Y<br>H199R<br>H199Q	H199Y<br>-<br>-	H199Y<br>-<br>-		
200	F200I				
201	V201M	V201M	V201M	V201M	
205	P205S<br>P205R	P205S<br>-	P205S<br>-		
206	L206W<br>L206F	L206W<br>-	L206W<br>-	L206W<br>-	L206W<br>-
209	A209S				
210	L210P				
213	G213V				
216	W216C				
217	E217G				
220	Q220R				
225	C225R				
227	L227R	L227R			
232	V232D	V232D	V232D	V232D	V232D
233	L233F				
234	-	-	-	A234D	A234D
237	Q237E<br>Q237H	Q237E<br>-	Q237E<br>Q237H	Q237E<br>Q237H	Q237E<br>Q237H
238	A238V				
239	G239R				
241	G241R				
243	M243L				
244	M244K				
248	R248T				
257	E257K				
258	R258G	R258G	R258G	R258G	
265	M265R	M265R	M265R	M265R	
277	W277R				
279	E279D				
281	M281T				

282	E282D				
285	I285F				
287	N287Y				
292	E292K				
293	L293M				
299	A299T				
301	Y301C				
305	F305V				
307	S307N				
309	A309T<br>A309G<br>A309D<br>A309V				
311	F311L	F311L	F311L	F311L	F311L
314	G314E<br>G314R<br>G314V	G314E<br>-<br>-	G314E<br>-<br>-	G314E<br>-<br>-	G314E<br>-<br>-
316	F316L				
317	V317A				
320	L320V<br>L320F	L320V<br>-	L320V<br>-	L320V<br>-	L320V<br>-
321	S321P				
322	V322A				
324	P324L				
325	Y325C				
327	L327R				
331	I331N				
333	L333F				
334	R334W<br>R334Q<br>R334L	R334W<br>R334Q<br>R334L	-<br>R334Q<br>R334L	-<br>R334Q<br>R334L	-<br>R334Q<br>R334L
336	I336K	I336K	I336K	I336K	
338	T338I<br>T338A	T338I	T338I	T338I	T338I
340	I340N				
341	S341P	S341P	S341P		
346	L346P	L346P	L346P	L346P	
347	R347P<br>R347H<br>R347C<br>R347L	R347P<br>R347H<br>-<br>-	R347P<br>R347H<br>-<br>R347L	R347P<br>R347H<br>-<br>R347L	-<br>R347H<br>-<br>R347L
348	M348V<br>M348T<br>M348K				
349	A349V	A349V	A349V	A349V	A349V
351	T351I<br>T351S				
352	R352Q<br>R352W<br>R352G	R352Q<br>R352W<br>-	R352Q<br>R352W<br>-	R352Q<br>R352W<br>-	R352Q<br>-<br>-

353	Q353H				
355	P355S				
356	W356S				
359	Q359R	Q359R	Q359R	Q359R	Q359R
360	T360R				
361	W361R		W361R		
364	S364P				
365	L365P				
369	N369Y				
373	D373E				
375	L375F				
378	Q378R				
379	E379K				
383	L383S				
388	T388M				
	**204** Missense Mutations	**54** Missense Mutations	**53** approved forTrikafta	**47** approved for Symdeko	**32** approved for Kalydeco

NBD1 Residue Numbers	CFMDB Missense Mutations	CFTR2 Missense Mutations	Eligible for Trikafta	Eligible for Symdeko	Eligible for Kalydeco
392	V392G<br>V392A				
394	M394R				
399	A399D<br>A399V				
403	E403D	-	E403D	E403D	
407	E407V				
416	N416S				
418	N418S				
424	G424S				
431	S431G				
439	P439S				
443	D443Y	D443Y	D443Y	D443Y	
444	I444T				
452	Q452P				
453	-	L453S	L453S		
455	A455E	A455E	A455E	A455E	A455E
456	V456A<br>V456F	V456A<br>-	V456A<br>V456F		
458	G458V	-	G458V		
463	G463V				
464	K464N				
466	S466L				
467	L467P	L467P			

468	L468P				
469	M469V				
470	M470V	M470V			
474	E474K	E474K	E474K		
480	G480S<br>G480C<br>G480D	-<br>-<br>-	-<br>G480C<br>-		
484	H484Y<br>H484R				
485	S485C<br>S485T				
491	C491R				
492	S492F	S492F			
493	Q493P<br>Q493R				
497	I497V				
498	M498I				
499	P499A				
501	T501A				
502	I502T<br>I502N	I502T			
504	E504Q				
506	I506L<br>I506S<br>I506T				
508	F508S<br>F508C	-<br>F508C	-<br>F508C	-<br>F508C	-<br>F508C
513	D513G	D513G			
515	Y515H				
516	R516G				
517	Y517C				
519	S519G				
520	V520F<br>V520I	V520F			
527	E527Q<br>E527G				
528	E528K<br>E528D				
529	D529H<br>D529G				
534	A534E				
536	K536E				
537	D537E				
539	I539T				
542	G542E				
544	G544S<br>G544SV				
548	L548Q				
549	S549N<br>S549R<br>S549I	S549N<br>S549R<br>-	S549N<br>S549R<br>-	S549N<br>S549R<br>-	S549N<br>S549R<br>-

550	G550R				
551	G551S<br>G551D	G551S<br>G551D	G551S<br>G551D	G551S<br>G551D	G551S<br>G551D
552	Q552K				
553	G553G<br>G553Q	-<br>-	G553G<br>G553Q	G553G<br>G553Q	G553G<br>G553Q
555	R555G				
556	I556V	-	I556V	I556V	I556V
558	L558S	L558S			
559	A559T<br>A559V<br>A559E	A559T<br>-<br>-			
560	R560K<br>R560T<br>R560S<br>R560G	R560K<br>R560T<br>R560S<br>-			
561	A561E	A561E			
562	V562I<br>V562L	V562I	V562I	V562I	V562I
563	Y563N<br>Y563D<br>Y563C	Y563N<br>Y563D<br>-	Y563N<br>-<br>-		
565	D565G				
566	A566T				
568	L568F				
569	Y569D<br>Y569H<br>Y569C	Y569D			
571	L571S				
572	D572N				
573	S573C				
574	P574H<br>P574S	P574H	P574H		
575	-	P575Y	P575Y	P575Y	
576	G576A	G576A			
577	Y577F				
579	D579G<br>D579Y<br>D579A	D579G<br>-<br>-	D579G<br>-<br>-	D579G<br>-<br>-	D579G<br>-<br>-
582	T582S<br>T582R<br>T582I				
586	I586V				
587	F587I				
588	E588V	E588V	E588V	E588V	
589	S589I<br>S589N	-<br>-	-<br>S589N	-<br>S589N	-<br>S589N
594	L594P				
595	M595T				
600	R600G				
601	I601F	I601F	I601F	I601F	

603	V603F				
604	T604S<br>T604I				
608	E608G				
609	H609R<br>H609L	H609R			
610	L610S				
613	A613T	A613T			
614	D614G<br>D614Y	D614G	D614G	D614G	
618	I618T	I618T	I618T	I618T	
619	L619S				
620	H620P<br>H620Q				
622	G622D	G622D	G622D	G622D	
628	G628R	G628R	G628R		
633	L633I<br>L633P				
636	L636P				
639	D639Y				
	**144** Missense Mutations in CFTR1	**38** Missense Mutations in CFTR2	**29** approved forTrikafta	**20** approved for Symdeko	**12** approved for Kalydeco

Supp Table S2: Mutations eligible for treatment with Trikafta, organized by their structural localization.

TMD1	NBD1
R74Q	S364P*
R74W	E403D*
R75Q	D443Y
G85E	L453S
E92K	A455E
Q98R	V456A
Y109N*	V456F
D110E	G463V*
D110H	E474K
E116K	G480C*
R117C	S492F
R117G	I502T
R117H	F508C
R117L	S549N
R117P	S549R
A120T	G551D
G126D	G551S
H139R	R553Q*
I148T	A554E*
M152V*	V562I
Y161D	Y563N
Y161S*	P574H
L165S	F575Y
R170H	G576A
I175V*	D579G
G178E	E588V
G178R*	S589N*
F191V	I601F
D192G	D614G
E193K	I618T
G194R	G622D
G194V	G628R
H199Y	
V201M	
P205S	
L206W	
V232D	
A234D*	
Q237E	
Q237H	
R258G	
M265R	
F311L	
G314E	

L320V
R334L
R334Q
I336K
T338I
S341P
L346P
R347H
R347L*
R347P
A349V
R352Q
R352W
Q359R
W361R*

*Mutations that are not listed in CFTR2 but are eligible for treatment with Trikafta.

Supp Table S3: Average of Predicted ΔΔG (kcal/mol) of CFTR mutants grouped by domain or presence of band C, using as template CFTR in an "open" conformation (6sms) or a "closed" conformation (5uak) by FoldX.

CFTR Structure	TMD1 (kcal/mol)	NBD1 (kcal/mol)
**6msm**	1.42 ± 2,17 (0.74 ± 0.75)[a]	3.92 ± 2,11
**5uak**	0.05 ± 1.33	2.04 ± 1.42
**7svd**	0.85 ± 1.73	3.24 ± 2.04
**7sv7**	1.28 ± 2.59	3.28 ± 1.27
	**Band C on WB (kcal/mol)**	**No band C (kcal/mol)**
**7svd**	0.06 ± 1,7	2.61 ± 1.81
**7sv7**	0.5 ± 1.3	3.1 ± 2.4

[a]excluding H199Y

Supp Table S4: Predicted ΔΔG Values ((ΔG(Mutant) − ΔG(WT)) (kcal/mol) for TMD1 and NBD1 Mutations Inclued in Trikafta Label Extension using FoldX

Mutation	5uak (Stability)	5uak (St dev)	6msm (Stability)	6msm (St dev)
NBD1 A455E	1.5763	0.7597	4.0114	0.64
NBD1 A554E	1.4002	0.1041	0.236	0.1067
NBD1 A559E	1.3211	0.1106	-3.5394	3.1282
NBD1 A559T	0.1632	0.0407	2.2631	0.2985
NBD1 D443Y	0.1062	0.0186	0.1818	0.04
NBD1 D579G	3.144	0.0201	7.5868	0.1749
NBD1 D614G	1.2118	0.0385	0.8793	0.1258
NBD1 E403D	n a		-0.0162	0.1036
NBD1 E474K	0.2788	0.408	4.4381	0.1402
NBD1 E588V	0.7775	0.0225	0.9543	0.0789
NBD1 F508C	2.5552	0.1453	3.8415	0.1022
NBD1 F575Y	0.4399	0.0035	1.0677	0.077
NBD1 G463V	4.9169	0.2451	9.6047	0.5372
NBD1 G480C	13.6899	0.2278	7.8955	0.4019
NBD1 G551D	0.6893	0.404	-0.264	0.3628
NBD1 G551S	-0.391	0.0302	0.7114	0.2572
NBD1 G576A	0.1315	0	1.194	0.2509
NBD1 G622D	8.3989	1.7263	9.0118	1.0153
NBD1 G628R	4.0647	0.3954	4.772	0.3108
NBD1 I502T	1.6982	0.2821	2.3214	0.0103
NBD1 I601F	4.1107	1.2853	7.4605	1.808
NBD1 I618T	2.8814	0.0278	3.0459	0.0228
NBD1 L453S	3.1488	0.3557	2.0766	0.1579
NBD1 L558S	2.7257	0.0171	3.4256	0.032
NBD1 L571S	3.9392	0.0358	4.0449	0.0177
NBD1 P574H	10.6698	1.6585	9.5251	2.8674
NBD1 R553Q	-0.2087	0.2402	0.2829	0.4342
NBD1 R560S	2.297	0.4656	2.9713	0.4293
NBD1 R560T	2.0602	0.7007	2.4006	0.4085
NBD1 S364P	4.3212	0.1224	1.8036	0.1496
NBD1 S492F	1.3467	1.7247	1.8168	2.201
NBD1 S549N	0.2327	0.0359	0.3806	0.0212
NBD1 S549R	0.5838	0.0294	2.3309	0.2354
NBD1 S589N	-0.8194	0.1589	-0.9522	0.0855
NBD1 V456A	2.6094	0.0119	3.5611	0.0135
NBD1 V456F	-1.5743	0.618	3.7544	0.2646
NBD1 V520T	1.5901	0.1844	1.0251	0.0281
NBD1 V562I	-1.2051	0.0389	0.1643	0.3743
NBD1 Y563C	2.5108	0.1269	4.0229	0.0235
NBD1 Y563D	3.6309	0.0848	5.8036	0.1669

NBD1 Y563N	2.6965	0.2326	4.36	0.0908
TMD1 A120T	1.8068	0.3823	0.7154	0.035
TMD1 A234D	1.2419	0.0213	1.1334	0.1443
TMD1 A349V	-0.4124	0.2572	-0.1847	0.3963
TMD1 D110E	-0.4889	0.0299	2.4576	0.1694
TMD1 D110H	-0.9811	0.0892	2.7622	0.3966
TMD1 D192G	-0.8954	0.0028	1.4327	0.3309
TMD1 E116K	0.2889	0.029	0.1381	0.3976
TMD1 E193K	-0.6951	0.197	-0.6568	0.0791
TMD1 E92K	0.8048	0.1889	0.687	0.1
TMD1 F191V	1.8287	0.0734	2.5149	0.1048
TMD1 F311L	1.5719	0.0749	1.521	0.0222
TMD1 G126D	-0.5228	0.0149	-0.7177	0.0054
TMD1 G178E	-1.7629	0.033	8.3889	0.5584
TMD1 G178R	-0.4146	0.082	8.7156	0.9432
TMD1 G194R	-0.6242	0.2414	0.9186	0.5597
TMD1 G194V	0.5744	0.0322	1.0694	0.092
TMD1 G314E	2.3742	1.2442	3.3991	1.501
TMD1 G85E	6.3274	1.6215	7.5798	3.9914
TMD1 G85V	5.5853	1.1353	6.1319	0.274
TMD1 H139R	0.0966	0.6405	0.3745	0.763
TMD1 H199Y	-0.5756	0.2405	6.8882	1.3511
TMD1 I148T	2.654	0.0099	1.8169	0.0292
TMD1 I175V	0.0036	0.0217	1.1325	0.0193
TMD1 I336K	1.0109	0.1153	1.0965	0.0164
TMD1 I507del	-0.0095	0.001	0.0019	0.009
TMD1 L165S	2.1245	0.0726	2.7067	0.0277
TMD1 L206W	0.9964	0.0922	0.3394	0.039
TMD1 L227R	1.9968	0.068	2.019	0.0409
TMD1 L320V	0.9638	0.0575	0.5538	0.1091
TMD1 L346P	3.8677	0.032	6.9786	0.1703
TMD1 M152V	3.0114	0.0998	1.2121	0.0411
TMD1 M265R	2.2353	0.1561	4.5782	0.5768
TMD1 P205S	2.2695	0.0433	1.7467	0.0267
TMD1 Q237E	1.2363	0.0683	2.6141	0.0103
TMD1 Q237H	1.6295	0.137	3.4684	0.1704
TMD1 Q359R	-0.0258	0.1564	0.5079	0.2225
TMD1 Q98R	1.6757	0.4027	0.2388	0.1037
TMD1 R117C	1.8401	0.0172	1.4479	0.0478
TMD1 R117G	2.0626	0.0171	1.4268	0.0272
TMD1 R117H	1.8541	0.0597	1.2424	0.0604
TMD1 R117L	-0.0864	0.2104	1.3621	0.0916
TMD1 R117P	1.2011	0.3126	-0.8031	0.0523

TMD1 R170H	0.2852	0.0343	1.6571	0.0604
TMD1 R258G	2.0202	0.5967	2.8737	0.1938
TMD1 R334L	-0.4534	0.0666	-1.003	0.2993
TMD1 R334Q	0.1935	0.036	-0.4469	0.351
TMD1 R347H	0.5532	0.4779	1.6534	0.887
TMD1 R347L	-1.3477	0.4136	-1.7737	0.7172
TMD1 R347P	5.0759	0.4681	3.7924	0.6891
TMD1 R352G	2.365	0.2348	2.4914	0.189
TMD1 R352Q	-0.5294	0.254	1.7828	0.0751
TMD1 R352W	-0.204	0.995	0.8487	0.2653
TMD1 R74Q	0.4641	0.097	0.9096	0.1308
TMD1 R74W	-1.2432	0.4668	-0.4917	0.173
TMD1 R75G	1.7596	0.1079	0.8242	0.1956
TMD1 R75Q	2.3412	0.0835	0.6495	0.4119
TMD1 S341P	-0.5575	0.0878	3.2122	0.0589
TMD1 T338I	-1.2658	0.0032	-0.765	0.0183
TMD1 V201M	-1.5268	0.0278	-0.6928	0.1311
TMD1 V232D	-0.0706	0.009	-0.5106	0.0021
TMD1 W361R	-0.4082	0.331	0.7489	0.2629
TMD1 W57G	3.9261	0.0576	5.4808	0.0557
TMD1 Y109N	1.9018	0.0458	2.2713	0.0103
TMD1 Y161D	4.5467	0.1193	5.0481	0.1072
TMD1 Y161S	2.9198	0.1086	4.143	0.1154

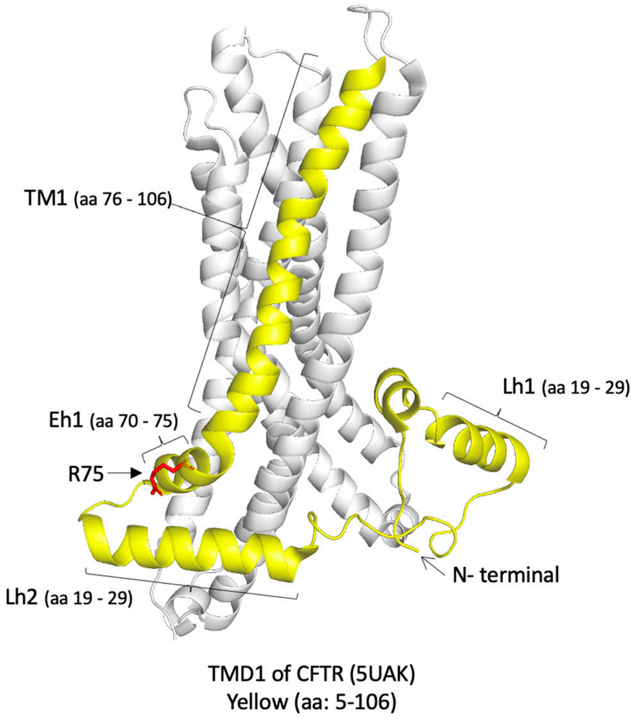

Supp Figure S1: N-terminal region of CFTR, with elbow helix region (Eh1), immediately ahead of TM1 and close to lasso motif 2 (Lh2). Part of TMD1 is also visible in grey. Residue R75 is represented in red.

Additional information on the mutations studied

In R75G, the positively charged arginine amino acid is replaced by the small and hydrophobic glycine. R75 forms a salt bridge with E56 and interacts with E60, and these bonds participate in a network linking TMD1 to the lasso lh2, ICL1, and ICL4 [2]. R75 has been identified [2] as a residue possibly contributing to a tight network of salt bridges that allows the accommodation of VX-809 at the identified TMD1 binding site, also influencing the allosteric coupling between TMD1 and NBD1.

In H139R, the basic amino acid histidine is changed to the basic amino acid arginine, and it was first described in an Italian patient [3]. The first attempt to rescue this mutant was made by Loo et al. [4] when they tested a novel quinazoline derivative to promote maturation and trafficking of misprocessed CFTR variants, although no effect on maturation was observed – probably due to the introduction of a bulky and highly charged residue at this position causing a large thermodynamic hurdle in the folding process. Data obtained after transient expression of COS-1 cells [5] show that H139R abolishes CFTR processing and decreases in the ability to transport Cl^-. Currently, H139R is part of the Trikafta label extension in the US.

I148T was first reported in a Canadian CF patient with pancreatic insufficiency [6]. The conserved I148 hydrophobic residue located in the ICL1 of CFTR (Figure 2) is replaced by the polar amino acid threonine. This mutation is considered a severe CF mutation because some patients bearing it, had classic CF with pancreatic insufficiency [6-8]. However, a study focused on understanding the different CF phenotypes of many individuals carrying I148T concluded that this mutation is associated with CF disease only if another mutation, 3199del6 (a deletion of ATAGTG from nucleotide 3199, located at CFTR exon 17a) is found in *cis*, forming a complex allele, while healthy adults who were either homozygous or heterozygous for I148T did not carry the deletion [9]. Although there is little doubt that 3199del6 is a deleterious CF mutation, the clinical significance of I148T in the absence of 3199del6 is unclear [10]. According to CFTR2, I148T is classified as non-CF-causing, but this variant is included in the Trikafta label extension granted by FDA [11].
Previous characterization of I148T reported maturation and anion conduction similar to CFTR WT when transiently expressed in HEK293 and COS-1 cells, respectively [5,12]. In primary cultures of nasal epithelial cells (HNE) obtained two heterozygous individuals for I148T [13], CFTR channel activity was reported as 87% of WT-CFTR. Other authors also reported normal protein processing and WT-like channel activity for I148T-CFTR in HEK293 cells but found that this variant suppresses the ability of CFTR to support HCO_3^- transport, which led them to hypothesize that I148T may contribute to disease through Cl^--coupled HCO_3^- altered transport [14].

D192G was first identified[15] in Slovenian CF patients. This is a drastic amino acid substitution, once the negatively charged aspartic acid, a well evolutionarily conserved residue [16], is replaced by the non-polar and small glycine residue. D192G is currently eligible for Trikafta, Symdeco, and Kalydeco.

G194R is a very rare mutation, reported in only 3 individuals worldwide according to CFTR2. In this case, the small and hydrophobic glycine is replaced by the basic arginine. This residue is localized in ICL2, where it is well embedded in the protein, in the small kink found in TM3. The first identification of G194R [17] was made in an African-American

female infant who also carries a 1341+1 G>A mutation and the 5T variant in intron 8. G194R is currently eligible for Trikafta, Symdeco, and Kalydeco.

H199Y is considered a severe CF-causing mutation, mainly because it is believed to be involved in inter-helix packing [2], since it is localized in a site of TM3 that is in close contact with TM1, where G85E, another severe CF-causing mutation, is localized. The presence of these mutations in TM1 and TM3 would disrupt the interaction between the two segments. G85E has indeed been shown to affect protein processing [18,19] and H199Y has probably a similar defect [20]. H199Y is currently eligible for Trikafta.

V201M changes a valine residue for a methionine in TM3. This mutation was first reported alone in a French patient with congenital bilateral absence of vas deferens (CBAVD) and then in Brazilian patients with CF[21]. Several studies have reported a link between CBAVD and the V201M mutation [22-24]. V201M is classified as a variant of unknown significance in CFTR2. This unknown significance can be assigned if variants have not yet been reported in a sufficient number of patients to allow proper classification or if they are found in a greater frequency in the healthy population [25]. The only study on the use of modulators in this variant [26] reports on the clinical benefit of Ivacaftor in a patient with the complex allele [R74W, V201M,D1270N]. Currently, V201M is eligible for Trikafta and Symdeko treatment [11,25].

In Q359R, the neutral polar glutamine is changed to the positively charged arginine. A recent clinical description of a heterozygous patient with Q359R/F508del shows typical CF sweat chloride values [16]. Q359R is currently eligible for treatment with Trikafta, Symdeko, and Kalydeco.

W361R mutation (c.1081T > A) was first detected in a French woman [27], and has also been reported in Spanish [28], Greek [29] and Swedish patients [30]. No information about this mutation is available in CFTR2. At the structural level, W361 is located within a class-II CF mutation hotspot that includes other mutations that induce variable disease severity. W361 is located in the TM6 helix, in a region where the elbow helix, TM1, TM3, and TM6 have close contacts forming a groove at the protein/membrane interface that can be stably occupied by lipid acyl chains [31]. W361R is currently eligible for Trikafta in the US.

In L558S, the hydrophobic leucine amino acid is replaced by polar and hydrophilic serine. L558S is registered in CFTR2 as a CF-causing mutation. This mutation affects the intermediate folding steps of NBD1 by disrupting the α-core compaction of nascent NBD1 during a critical window of folding [32]. Currently, L558S is not eligible for any of the FDA-approved modulators.

Residue A559 is located in an α-helical subdomain of NBD1 in a large hotspot of missense mutations at the NBD1: ICL4 interface [33]. Alanine is a small hydrophobic amino acid that is replaced by the acidic and hydrophilic glutamic acid and in the case of A559T by the polar and hydrophilic threonine. A559T has so far been found mainly in patients with CF of African American origin [34-36] and is registered in CFTR2 as a CF-causing mutation affecting 85 patients. Currently, none of the mutations in residue A559 (A559E, A559T and A559V) is eligible for treatment with FDA-approved modulators.

R560T is one of the most common CFTR mutations, particularly in the Northern Irish population [36]. Due to its prevalence, this mutation is well characterized. It lies in α-helix (H5) within the ATP-binding cassette α-subdomain of NBD1, in a hot spot for mutations between the signature (LSGGQ) and Walker B motifs [37]. According to previous reports [37,38] the side chain of R560 is partially buried in the tertiary structure of NBD1, and the mutation R560T likely disrupts the interactions of R560 with neighboring residues and therefore impacts the folding of NBD1. When expressed in COS-7[39] or BHK cells [37], R560T also abolished CFTR processing. The reverting mutation G550E, which can rescue the expression and function of the cell surface of F508del-CFTR, also failed to revert the defect caused by R560T [37]. This mutation is not eligible for treatment with any of the FDA-approved modulators. Other mutations in this residue – e.g. R560S and R560G – also cause severe disease, highlighting its importance for the global structure of CFTR.

References

1. Liu, F.; Zhang, Z.; Csanady, L.; Gadsby, D.C.; Chen, J. Molecular Structure of the Human CFTR Ion Channel. *Cell* **2017**, *169*, 85-95 e88, doi:10.1016/j.cell.2017.02.024.
2. Baatallah, N.; Elbahnsi, A.; Mornon, J.P.; Chevalier, B.; Pranke, I.; Servel, N.; Zelli, R.; Decout, J.L.; Edelman, A.; Sermet-Gaudelus, I.; et al. Pharmacological chaperones improve intra-domain stability and inter-domain assembly via distinct binding sites to rescue misfolded CFTR. *Cell Mol Life Sci* **2021**, doi:10.1007/s00018-021-03994-5.
3. Ferec, C.; Novelli, G.; Verlingue, C.; Quere, I.; Dallapiccola, B.; Audrezet, M.P.; Mercier, B. Identification of six novel CFTR mutations in a sample of Italian cystic fibrosis patients. *Mol.Cell Probes.* **1995**, *9*, 135-137.
4. Loo, T.W.; Bartlett, M.C.; Clarke, D.M. Rescue of DeltaF508 and other misprocessed CFTR mutants by a novel quinazoline compound. *Mol Pharm* **2005**, *2*, 407-413, doi:10.1021/mp0500521.
5. Seibert, F.S.; Jia, Y.; Mathews, C.J.; Hanrahan, J.W.; Riordan, J.R.; Loo, T.W.; Clarke, D.M. Disease-associated mutations in cytoplasmic loops 1 and 2 of cystic fibrosis transmembrane conductance regulator impede processing or opening of the channel. *Biochemistry* **1997**, *36*, 11966-11974.
6. Bozon, D.; Zielenski, J.; Rininsland, F.; Tsui, L.C. Identification of four new mutations in the cystic fibrosis transmembrane conductance regulator gene: I148T, L1077P, Y1092X, 2183AA- ->G. *Hum.Mutat.* **1994**, *3*, 330-332.
7. Castaldo, G.; Rippa, E.; Raia, V.; Salvatore, D.; Massa, C.; de Ritis, G.; Salvatore, F. Clinical features of cystic fibrosis patients with rare genotypes. *J Med Genet* **1996**, *33*, 73-76, doi:10.1136/jmg.33.1.73.
8. Population variation of common cystic fibrosis mutations. The Cystic Fibrosis Genetic Analysis Consortium. *Hum Mutat* **1994**, *4*, 167-177, doi:10.1002/humu.1380040302.
9. Rohlfs, E.M.; Zhou, Z.; Sugarman, E.A.; Heim, R.A.; Pace, R.G.; Knowles, M.R.; Silverman, L.M.; Allitto, B.A. The I148T CFTR allele occurs on multiple haplotypes: a complex allele is associated with cystic fibrosis. *Genet Med* **2002**, *4*, 319-323, doi:10.1097/00125817-200209000-00001.
10. Monaghan, K.G.; Highsmith, W.E.; Amos, J.; Pratt, V.M.; Roa, B.; Friez, M.; Pike-Buchanan, L.L.; Buyse, I.M.; Redman, J.B.; Strom, C.M.; et al. Genotype-phenotype correlation and frequency of the 3199del6 cystic fibrosis mutation among I148T carriers: results from a collaborative study. *Genet Med* **2004**, *6*, 421-425, doi:10.1097/01.GIM.0000139507.20179.3A.
11. Salvatore, D.; Pepe, A.; Carnovale, V.; Majo, F.; Padoan, R.; Quattrucci, S.; Salvatore, M.; Taruscio, D.; Amato, A.; Ferrari, G.; et al. Elexacaftor/tezacaftor/ivacaftor for CFTR variants giving rise to diagnostic uncertainty: Personalised medicine or over-medicalisation? *J Cyst Fibros* **2022**, *21*, 544-548, doi:10.1016/j.jcf.2021.09.011.
12. Caputo, A.; Hinzpeter, A.; Caci, E.; Pedemonte, N.; Arous, N.; Di Duca, M.; Zegarra-Moran, O.; Fanen, P.; Galietta, L.J. Mutation-specific potency and efficacy of cystic fibrosis transmembrane conductance regulator chloride

channel potentiators. *J Pharmacol Exp Ther* **2009**, *330*, 783-791, doi:jpet.109.154146
13. Terlizzi, V.; Castaldo, G.; Salvatore, D.; Lucarelli, M.; Raia, V.; Angioni, A.; Carnovale, V.; Cirilli, N.; Casciaro, R.; Colombo, C.; et al. Genotype-phenotype correlation and functional studies in patients with cystic fibrosis bearing CFTR complex alleles. *J Med Genet* **2017**, *54*, 224-235, doi:10.1136/jmedgenet-2016-103985.
14. Choi, J.Y.; Muallem, D.; Kiselyov, K.; Lee, M.G.; Thomas, P.J.; Muallem, S. Aberrant CFTR-dependent HCO3- transport in mutations associated with cystic fibrosis. *Nature* **2001**, *410*, 94-97, doi:10.1038/35065099.
15. Audrezet, M.P.; Canki-Klain, N.; Mercier, B.; Bracar, D.; Verlingue, C.; Ferec, C. Identification of three novel mutations (457 TAT-->G, D192G, Q685X) in the Slovenian CF patients. *Hum Genet* **1994**, *93*, 659-662, doi:10.1007/BF00201566.
16. Zybert, K.; Wozniacki, L.; Tomaszewska-Sobczynska, A.; Wertheim-Tysarowska, K.; Czerska, K.; Oltarzewski, M.; Sands, D. Clinical characteristics of rare CFTR mutations causing cystic fibrosis in Polish population. *Pediatr Pulmonol* **2020**, *55*, 2097-2107, doi:10.1002/ppul.24823.
17. Keiles, S.; Kammesheidt, A. Identification of CFTR, PRSS1, and SPINK1 mutations in 381 patients with pancreatitis. *Pancreas* **2006**, *33*, 221-227, doi:10.1097/01.mpa.0000232014.94974.75.
18. Van Goor, F.; Yu, H.; Burton, B.; Hoffman, B.J. Effect of ivacaftor on CFTR forms with missense mutations associated with defects in protein processing or function. *J Cyst Fibros* **2014**, *13*, 29-36, doi:10.1016/j.jcf.2013.06.008.
19. Decaestecker, K.; Decaestecker, E.; Castellani, C.; Jaspers, M.; Cuppens, H.; De Boeck, K. Genotype/phenotype correlation of the G85E mutation in a large cohort of cystic fibrosis patients. *Eur Respir J* **2004**, *23*, 679-684, doi:10.1183/09031936.04.00014804.
20. Zhang, Z.; Chen, J. Atomic Structure of the Cystic Fibrosis Transmembrane Conductance Regulator. *Cell* **2016**, *167*, 1586-1597 e1589, doi:10.1016/j.cell.2016.11.014.
21. Bernardino, A.L.; Ferri, A.; Passos-Bueno, M.R.; Kim, C.E.; Nakaie, C.M.; Gomes, C.E.; Damaceno, N.; Zatz, M. Molecular analysis in Brazilian cystic fibrosis patients reveals five novel mutations. *Genet Test* **2000**, *4*, 69-74, doi:10.1089/109065700316516.
22. Wu, C.C.; Alper, O.M.; Lu, J.F.; Wang, S.P.; Guo, L.; Chiang, H.S.; Wong, L.J. Mutation spectrum of the CFTR gene in Taiwanese patients with congenital bilateral absence of the vas deferens. *Hum Reprod* **2005**, *20*, 2470-2475, doi:10.1093/humrep/dei077.
23. Danziger, K.L.; Black, L.D.; Keiles, S.B.; Kammesheidt, A.; Turek, P.J. Improved detection of cystic fibrosis mutations in infertility patients with DNA sequence analysis. *Hum Reprod* **2004**, *19*, 540-546, doi:10.1093/humrep/deh134.
24. Boudaya, M.; Fredj, S.H.; Haj, R.B.; Khrouf, M.; Bouker, A.; Halouani, L.; Messaoud, T. Cystic fibrosis transmembrane conductance regulator mutations and polymorphisms associated with congenital bilateral absence of vas deferens in a restricted group of patients from North Africa. *Ann Hum Biol* **2012**, *39*, 76-79, doi:10.3109/03014460.2011.642892.

25. Raraigh, K.S.; Lewis, M.H.; Collaco, J.M.; Corey, M.; Penland, C.M.; Stephenson, A.L.; Rommens, J.M.; Castellani, C.; Cutting, G.R. Caution advised in the use of CFTR modulator treatment for individuals harboring specific CFTR variants. *J Cyst Fibros* **2022**, *21*, 856-860, doi:10.1016/j.jcf.2022.04.019.
26. Arora, K.; Yang, F.; Brewington, J.; McPhail, G.; Cortez, A.R.; Sundaram, N.; Ramananda, Y.; Ogden, H.; Helmrath, M.; Clancy, J.P.; et al. Patient personalized translational tools in cystic fibrosis to transform data from bench to bed-side and back. *Am J Physiol Gastrointest Liver Physiol* **2021**, *320*, G1123-G1130, doi:10.1152/ajpgi.00095.2021.
27. Bienvenu, T.; Hubert, D.; Fonknechten, N.; Dusser, D.; Kaplan, J.C.; Beldjord, C. Unexpected inactivation of acceptor consensus splice sequence by a -3 C to T transition in intron 2 of the CFTR gene. *Hum.Genet.* **1994**, *94*, 65-68.
28. Alonso, M.J.; Heine-Suner, D.; Calvo, M.; Rosell, J.; Gimenez, J.; Ramos, M.D.; Telleria, J.J.; Palacio, A.; Estivill, X.; Casals, T. Spectrum of mutations in the CFTR gene in cystic fibrosis patients of Spanish ancestry. *Ann Hum Genet* **2007**, *71*, 194-201, doi:10.1111/j.1469-1809.2006.00310.x.
29. Kanavakis, E.; Tzetis, M.; Antoniadi, T.; Traeger-Synodinos, J.; Doudounakis, S.; Adam, G.; Matsaniotis, N.; Kattamis, C. Mutation analysis of ten exons of the CFTR gene in Greek cystic fibrosis patients: characterization of 74.5% of CF alleles including one novel mutation. *Hum.Genet.* **1995**, *96*, 364-366.
30. Strandvik, B.; Bjorck, E.; Fallstrom, M.; Gronowitz, E.; Thountzouris, J.; Lindblad, A.; Markiewicz, D.; Wahlstrom, J.; Tsui, L.C.; Zielenski, J. Spectrum of mutations in the CFTR gene of patients with classical and atypical forms of cystic fibrosis from southwestern Sweden: identification of 12 novel mutations. *Genet Test* **2001**, *5*, 235-242, doi:10.1089/10906570152742290.
31. Gregory, R.J.; Rich, D.P.; Cheng, S.H.; Souza, D.W.; Paul, S.; Manavalan, P.; Anderson, M.P.; Welsh, M.J.; Smith, A.E. Maturation and function of cystic fibrosis transmembrane conductance regulator variants bearing mutations in putative nucleotide-binding domains 1 and 2. *Mol Cell Biol* **1991**, *11*, 3886-3893.
32. Shishido, H.; Yoon, J.S.; Yang, Z.; Skach, W.R. CFTR trafficking mutations disrupt cotranslational protein folding by targeting biosynthetic intermediates. *Nat Commun* **2020**, *11*, 4258, doi:10.1038/s41467-020-18101-8.
33. Callebaut, I.; Hoffmann, B.; Lehn, P.; Mornon, J.P. Molecular modelling and molecular dynamics of CFTR. *Cell Mol Life Sci* **2017**, *74*, 3-22, doi:10.1007/s00018-016-2385-9.
34. Stewart, C.; Pepper, M.S. Cystic Fibrosis in the African Diaspora. *Ann Am Thorac Soc* **2017**, *14*, 1-7, doi:10.1513/AnnalsATS.201606-481FR.
35. Vouk, K.; Strmecki, L.; Liovic, M.; Kopriva, S.; Micetic-Turk, D.; Komel, R. Mutational analysis of 30 Slovenian cystic fibrosis patients compared to known Slovenian and European CF mutation spectra. *Pflugers Arch* **2000**, *439*, R63-R65.
36. Castellani, C.; Cuppens, H.; Macek, M., Jr.; Cassiman, J.J.; Kerem, E.; Durie, P.; Tullis, E.; Assael, B.M.; Bombieri, C.; Brown, A.; et al. Consensus on the use and interpretation of cystic fibrosis mutation analysis in clinical practice. *J Cyst Fibros* **2008**, *7*, 179-196, doi:10.1016/j.jcf.2008.03.009.

37. Roxo-Rosa, M.; Xu, Z.; Schmidt, A.; Neto, M.; Cai, Z.; Soares, C.M.; Sheppard, D.N.; Amaral, M.D. Revertant mutants G550E and 4RK rescue cystic fibrosis mutants in the first nucleotide-binding domain of CFTR by different mechanisms. *Proc Natl Acad Sci U S A* **2006**, *103*, 17891-17896, doi:0608312103
38. Lewis, H.A.; Zhao, X.; Wang, C.; Sauder, J.M.; Rooney, I.; Noland, B.W.; Lorimer, D.; Kearins, M.C.; Conners, K.; Condon, B.; et al. Impact of the deltaF508 mutation in first nucleotide-binding domain of human cystic fibrosis transmembrane conductance regulator on domain folding and structure. *The Journal of biological chemistry* **2005**, *280*, 1346-1353, doi:10.1074/jbc.M410968200.
39. Lopes-Pacheco, M.; Sabirzhanova, I.; Rapino, D.; Morales, M.M.; Guggino, W.B.; Cebotaru, L. Correctors Rescue CFTR Mutations in Nucleotide-Binding Domain 1 (NBD1) by Modulating Proteostasis. *Chembiochem* **2016**, *17*, 493-505, doi:10.1002/cbic.201500620.

Numbering as cited in the supplementary information; cited with independent numbering in the main text.
